# Supplementary material for: Institutions matter: The role of institutions in the relationship between decision-making power and contraceptive autonomy
Source: PLOS Glob Public Health. 2024 Nov 13;4(11):e0003913. doi: 10.1371/journal.pgph.0003913 (PMC11560016; doi:10.1371/journal.pgph.0003913)
Supplement: Attachment — Submitted filename: PGPH-D-23-02195_R1_reviewer.pdf [file pgph.0003913.s003.pdf]

PLOS Global Public Health

‘Institutions matter: The role of institutions in the relationship between decision-making power and contraceptive autonomy’

--Manuscript Draft--

**Manuscript Number:**	PGPH-D-23-02195R1
**Article Type:**	Research Article
**Full Title:**	‘Institutions matter: The role of institutions in the relationship between decision-making power and contraceptive autonomy’
**Short Title:**	Institutions, decision-making power and contraceptive autonomy.
**Corresponding Author:**	Laura Rossouw<br>University of the Witwatersrand<br>Johannesburg, SOUTH AFRICA
**Order of Authors:**	Laura Rossouw
	Adeola Oyenubi
**Keywords:**	Decision-making power; autonomy; contraception; empowerment; Gender; Institutions
**Abstract:**	Progress in the development of modern contraception is recognized as one of the 10 great public health achievements of the 20th century. Several academic papers have shown that the distribution of household decision-making power has a positive influence on women’s contraceptive autonomy. This paper considers the role of the social or institutional environment (as captured by religion and place of residence) in ameliorating or contributing to this relationship. Our study focuses on the context of Nigeria, a country with diverse gender norms and religious practices, often determined by geographic location. For example, Western common law is more prevalent in the Southern Nigeria, while Sharia Law is largely practiced in the North of the country. The analysis uses the Performance and Monitoring for Action (PMA) dataset (2019-2020), and analysis is done using logistic regressions. we control for selection on observables using the Gradient boosted model. We find evidence that the relationship between household decision-making power and contraceptive autonomy is mediated by institutions (city and religion). More specifically, we show that in Lagos – a state characterised by the increasing empowerment of women – women have more contraceptive autonomy as they gain decision-making power in the household. The opposite is true in Kano - household decision-making power has no bearing on contraceptive autonomy.
**Additional Information:**	
**Question**	**Response**
**Financial Disclosure**<br><br>Enter a financial disclosure statement that describes the sources of funding for the work included in this submission and the role the funder(s) played. This includes grants and any commercial funding of the work or authors.<br><br>This statement will be typeset if the manuscript is accepted for publication.	No funding was received to write this article.

Powered by Editorial Manager® and ProduXion Manager® from Aries Systems Corporation

Please review the submission guidelines and the instructions link below for detailed requirements and guidance.	
**Competing Interests**<br><br>On behalf of all authors, disclose any competing interests that could be perceived to bias this work.<br><br>This statement will be typeset if the manuscript is accepted for publication.<br><br>*Please review the instructions link below and PLOS Global Public Health's competing interests policy to determine what information must be disclosed at submission.*	The authors report that there are no competing interests to declare.
**Data Availability**<br><br>Before publication, Authors are required to make fully available and without restriction all data underlying their findings. Please see our PLOS Data Policy page for detailed information on this policy.<br><br>A **Data Availability Statement**, detailing where the data can be accessed, is required at first submission. Insert your Data Availability Statement in the box below.<br><br>Please see the data reporting section of our submission guidelines for instructions on what you need to include in your Data Availability Statement.<br><br>This statement will be typeset if the manuscript is accepted for publication.<br><br>*PLOS allows rare exemptions to address*	The authors use the Performance Monitoring for Action (PMA) data set. Given that this is third party data, the authors are not allowed to share the data set. However, it can be easily downloaded from the PMA website, by registering and providing a brief description of the intended use for the data. The exact location for registering is here: https://www.pmadata.org/data/request-access-datasets. Please note that the current analysis can be completed replicated using this data set and following the steps outlined in the methodology. The authors had no special privileges pertaining to this data set.

legal and ethical concerns. If you have legal or ethical restrictions, please use the box below to detail these in full sentences for the Journal team to consider.	

Powered by Editorial Manager® and ProduXion Manager® from Aries Systems Corporation

Manuscript

Click here to access/download;Manuscript;Manuscript_November 2023.docx

Title: 'Institutions matter: The role of institutions in the relationship between decision-making power and contraceptive autonomy'

Short title: Institutions, decision-making power and contraceptive autonomy.

Corresponding author: Laura Rossouw, School of Economics and Finance, University of the Witwatersrand, Johannesburg, South Africa. http://orcid.org/0000-0001-7697-9567. Email: laura.rossouw@wits.ac.za; Telephone:+27 (0)11 717 9999

Adeola Oyenubi, School of Economics and Finance, University of the Witwatersrand, Johannesburg, South Africa. http://orcid.org/0000-0001-6068-1723

Abstract:

As we are nearly 25 years into the 21st Century, this opening sentence feels dated. Also, this sentence/concept is not used in the manuscript.

Progress in the development of modern contraception is recognized as one of the 10 great public health achievements of the 20th century. Several academic papers have shown that the distribution of household decision-making power has a positive influence on women's contraceptive autonomy. This paper considers the role of the social or institutional environment (as captured by religion and place of residence) in ameliorating or contributing to this relationship. Our study focuses on the context of Nigeria, a country with diverse gender norms and religious practices, often determined by geographic location. For example, Western common law is more prevalent in the Southern Nigeria, while Sharia Law is largely practiced in the North of the country. The analysis uses the Performance and Monitoring for Action (PMA) dataset (2019-2020), and we control for selection on observables using the Gradient boosted model. We find evidence that the relationship between household decision-making power and contraceptive autonomy is mediated by institutions (city and religion), but only for women with some decision-making power in their household. Among women with no decision-making power in the household, institutions do not play a role in their contraceptive autonomy. This finding highlights the potential severity of household level disempowerment of women.

Keywords: Decision-making power; Autonomy; Contraception; Empowerment; Gender

Word count: 5904 words

Introduction

The fertility rate in Nigeria has been steadily declining, from an average of 6.4 births per woman in 1960 to 5.3 in 2019. Despite the incremental decline, this fertility rate is almost double the average in lower-middle other lower middle income countries (The World Bank, 2022a). However, with a population of over 206 million (The World Bank, 2022b), Nigeria is a large and diverse country with 36 states, several ethnic groups and different socio-cultural identities (Adebowale, 2019), and average reproductive trends may misconstrue state-level tendencies. Most notably, Nigerian fertility rates are reportedly higher in the Northern states than the Southern states (National Population Commission, 2013). A key predictor of fertility includes access to modern contraception (Bongaarts, 2017). In 2013, access to contraception by women in Nigeria was still heavily skewed to the Southern States at 44 percent, compared to 12 percent in the Northern States (Rettig, Fick, & Hijmans, 2020).

consider adding a definition of "access to contraceptive methods"

Access to modern contraceptive methods is a function of several structural, cultural, and socio-economic factors, including women's empowerment and household bargaining power (Do & Kurimoto, 2012; Nkoka, Lee, Chuang, & Chuang, 2021; F. M. OlaOlorun & Hindin, 2014; Patrikar, Basannar, & Sharma, 2014; Yaya, Uthman, Ekholuenetale, & Bishwajit, 2018). Contraceptive knowledge, access and use are shaped by personal factors (like autonomy, attitudes and feelings about contraception), community influences (Mutumba, Wekesa, & Stephenson, 2018), and the surrounding socioeconomic and cultural environment (i.e. informal institutions) (Brown & Eisenberg, 1995). The role of the cultural environment and informal institutions in shaping women's empowerment and household bargaining power, and therefore, access to modern contraception is an area that has not

been evaluated extensively. In this article, we investigate the relationship between informal institutions in Nigeria (captured by the respondent's city and religion) and contraceptive autonomy, through the impact of these institutions on women's household decision-making power. Contraceptive autonomy refers to the ability of women to access their contraception of choice, but also includes the choice to

to access or to use?

opt out of using any contraception.

According to North, institutions are 'humanly devised constraints that structure human interactions. They are made up of formal constraints (rules, laws, constitutions), informal constrains (norms of behaviour, convention, and self-imposed codes of conduct), and their enforcement characteristics' (North, 1991). The expectation that institutional environments or context can influence the relationship between women's empowerment and contraceptive autonomy is derived from the field of psychology (V. Chirkov, 2017; V. I. Chirkov, Ryan, & Sheldon, 2010; Park & Chirkov, 2020). Specifically, the literature on the relationship between culture, self, and autonomy suggests that culture (or context) plays a key role in determining the potential for autonomy (V. Chirkov, 2017). Culture influences the definition of self, and by doing so sets the boundaries for a socially acceptable level of self-determination or autonomy (Park & Chirkov, 2020). The implication is that culture as a context is integral to one's construction of self and therefore autonomy. Therefore, if we accept the premise that women empowerment or autonomy influences the choice to use or not use contraceptives, and

context- or culture-specific

empowerment is context or culture specific, then one should expect that context will mediate the relationship between empowerment and contraceptive use. If institutions determine the boundaries of general autonomy and decision-making power, we expect that institutions will affect contraceptive autonomy.

In our analysis, institutions are defined as social norms, culture, and religion as determined by respondents' geo-location. In Nigeria, women's geographical location is likely to determine their ethnic group, lifestyle, and often religion (Adebowale, 2019). Our analysis is restricted to two Nigerian cities, namely Kano and Lagos. Kano is situated in northern Nigeria where Sharia Law is largely practiced. Lagos is situated in southern Nigeria where western common law is more prevalent (Elaigwu & Galadima, 2003; Kendhammer, 2013; Ostien & Dekker, 2010). The Hausa/Fulani ethnic groups often reside in the North of the country, while the Igbo and Yoruba tribes reside in the South (National Population Commission, 2015). In their national and subnational evaluation of female empowerment in Nigeria, Rettig *et al.* find that while female empowerment increased overall in Nigeria since the 1990s, this is largely driven by improvements in the South, with the Northern Nigerian states remaining relatively unchanged.

Differences in institutions between northern and southern Nigeria can arguably be dated back to pre-colonial days. Prior to the arrival of the British, Arabic traders ventured into the northern Nigeria (Fadiran, Sarr, & Fedderke, 2017). Consequently, Hausa people occupying northern Nigeria have a cultural heritage predominated by Arabic influence. The political system, for example, was based on Islamic law (Fadiran et al., 2017). Western influence and religion is more strongly represented in heritage and the way of life in Southern Nigeria. For example, transatlantic slave trade in Southern Nigeria pre-dated 1790 (Ojo, 2008) while western influence in form of Christian missionaries in southern Nigeria dates back to 1841 (Nwadialor, 2013). This difference in history affects present reality and in part explains differences in institutional environments in present day Nigeria. The analysis of these two cities situated in different states allows us to investigate the potential role of institutions and social norms in the relationship between household decision-making power and contraceptive autonomy.

Methods

Data

For this analysis, we used the Performance Monitoring for Action Nigeria Phase 1 Household and Female Survey Dataset, which focused on contraception, reproductive health, and family planning. The specific Nigerian dataset was chosen as it also explores women's autonomy. The data was sampled using a stratified cluster design with urban-rural strata, based on the National Population Commission's master sampling frame. Data was collected during December 2019 and January 2020. The final sample collected consists of 1122 women in Kano and 1469 women in Lagos (Centre for Research, Bayero University Kano (BUK), & The Bill & Melinda Gates Institute for Population and Reproductive Health at The Johns Hopkins Bloomberg School of Public Health, 2020). We restricted the sample to women who were married or living in a cohabiting relationship, so as do gauge women's contraceptive autonomy within the context of a sexually active relationship and household. As a result, we have a sample of 788 and 865 in Kano and Lagos respectively.

Variables

Dependent variable: Contraceptive autonomy

Our definition of contraceptive autonomy coincides with the modern perspective on reproductive decision-making. Woldemicael writes that the definition of women's autonomy in reproductive decisions shifted after the 1994 International Conference on Population and Development, from joint

?? not sure what the "nineties" means here

or interdependent decision-making with a spouse during the nineties, to emphasizing the role of woman's individual choice (Woldemicael, 2009). This follows the argument of Senderowicz (2020), who noted that non-autonomous use of contraception is also a negative outcome when it comes to autonomy and contraceptive use. By exploring contraceptive autonomy, rather than contraceptive use, we are able to capture the nuanced and dynamic nature of contraceptive choice.

Contraceptive autonomy was captured using the questions 'Would you say that using contraception is mainly your decision' and 'Would you say that not using contraception is mainly your decision'. For both of these questions, respondents could answer that the decision was either a) mainly theirs, b) the decision of their husband/partner, c) a joint decision or d) somebody else made the decision. Using this information, three variables of contraceptive autonomy was created.

- Lack of contraceptive autonomy (LAC_1): A binary variable equal to one if the respondent is not involved with their contraceptive choices. Either their husband/partner or somebody else made the decision for their contraceptive use or non-use.
- Lack of autonomy in choice to use contraception (LAC_2): A binary variable equal to one if the respondent is not the main decision-maker with regards to using contraception. Either their husband/partner or somebody else made the decision for their contraceptive use.
- Lack of autonomy in choice to not use contraception (LAC_3): A binary variable equal to one if the respondent is not the main decision-maker with regards to not using contraception. Either their husband/partner or somebody else made the decision for their contraceptive non-use.

We include the final two outcome variables in our analysis to test for the possibility that the factors related to non-autonomous use may be different from non-autonomous non-use of contraception.

Independent variable of interest: Household decision-making power

Household decision-making power was established using a set of six questions. Respondents were asked whom in the household makes decisions around (1) large household purchases, (2) household purchases for daily needs, (3) medical treatment for the respondent, (4) buying clothes for the respondent, (5) the spending of the respondent's earnings, or (6) how the respondent's partner's earnings are spent. For each of these questions, respondents could answer that they either a) made the decision themselves, b) their husband or partner made the decision, c) they made the decision with their partner jointly, or d) someone else decided.

Using this data, two decision-making categories were created:

- Sole or joint decision-maker. A binary variable equal to one if respondents were involved in decision-making (either solely or jointly) in at least three of the six questions listed above, and zero if not.
- Non-decision-maker: A binary variable equal to one if the respondent answered that their husband or partner, or someone else made their decisions in at least three of the six questions listed above, and zero if not.

Covariates

We control for several covariates that explain contraceptive autonomy, including marital status ('Currently married', 'Currently living with man') and whether it is polygamous, duration of co-habiting, age, possession of health insurance, ethnicity (Hausa, Igbo, Yoruba, and others), and whether

the respondent is the head of household. The respondent's economic and financial position is captured by including a dummy variable equal to one if the respondent has savings, land ownership, earning status (are they renumerated in 'Cash', 'Cash and kind', 'In-kind', 'Not paid' and 'Not working') and the respondents household wealth quintile.

Our first institutional variable is state, which is a binary variable equal to one if the respondent resides in Lagos and zero if they reside in Kano. Our second institutional variable is a categorical religion variable (Christian, Islamic, Other).

We also control for variables that capture autonomy relating to sexual behaviour in general.

- Perceived lack of sexual autonomy: A summary index ranging from zero to four, with a value of one every time a respondent answered yes to the following 'If I refuse sex with my husband/partner, he may stop supporting me', 'If I refuse sex with my husband/partner, he may force me to have sex', 'If I refuse sex with my husband/partner, he may physically hurt me', 'If I show my partner that I want to have sex, he may consider me promiscuous'.
- Sexual autonomy: A summary index ranging from zero to four, with a value of one every time a respondent answered yes to the following 'I am confident I can tell my husband/partner when I want to have sex', 'I am able to decide when to have sex', 'If I do not want to have sex, I can tell my husband/partner', 'If I do not want to have sex, I am capable of avoiding it'.
- Knowledge of contraception: A summary index ranging from zero to 16, with a value of one every time a respondent answered that they have heard of the following contraceptive methods: 'female sterilization', 'male sterilization', 'implants', 'IUD', 'injectables', 'birth control pills', 'emergency contraception', 'male condoms', 'female condoms', 'diaphragm', 'foam or

jelly as a contraceptive method', 'standard days method or cycle beads', 'LAM', 'rhythm', 'withdrawal' and 'others'.

Data Analyses

Our analysis consists of two parts. Firstly, we use logistic regression to estimate the relationship between household decision-making power and the level of autonomy in contraceptive use. However, since this approach does not account for differences in distribution of covariates between those who have autonomy in household decision-making and those who do not, we consider a model that accounts for the difference in characteristics. We therefore consider an approach that has causal interpretation under the Conditional Independence Assumption (CIA) (Rosenbaum & Rubin, 1983).
sentence needs clarification
However, we note that causal interpretation is only valid under the CIA, since we cannot control for unobserved covariates, this approach can be interpreted as relationship between the variables of interest while controlling for selection based on observed covariates. This approach utilizes the propensity score, or the probability of having general autonomy given observed covariates. The scores are then used to achieve balance in the distribution of covariates (by weighting or matching) so that the resulting estimate is not biased by the difference in distribution of covariates across groups.

A propensity score model specifies the relationship between a set of pre-treatment covariates and a treatment of interest, which in our analysis is household decision-making power. While in general any model relating a set of covariates to group assignment can be used to estimate the propensity scores (e.g., logistic regression), it has been found that misspecification of the propensity score model can lead to bias in the resulting effect estimate (Kosuke Imai & Ratkovic, 2014; Zhao, 2008). To solve this problem, researchers have started using non-parametric, data-driven optimization algorithms to

estimate propensity scores (Collier, Leite, & Zhang, 2021; Diamond & Sekhon, 2013; McCaffrey, Ridgeway, & Morral, 2004).

One such method is the Gradient boosted model (GBM) approach (Friedman, 2001). GBM is a machine learning approach that can be used to fit a nonlinear surface and predict group assignment while reducing bias that may arise from misspecification of the propensity score model. This is because GBM is a non-parametric approach that allows flexible, non-linear relationships in estimating propensity scores. The general idea is to optimize balance in the distribution of covariates across groups by selecting the iteration of the GBM that achieves the best balance (Ridgeway et al., 2021). The main advantage of this approach is that it captures non-linear effects and interactions terms without prior knowledge of the model. By automatically considering higher order terms this approach makes CIA more plausible. GBM has been shown to be less biased than parametric models (Choi, Wang, Michalek, & Gelfond, 2019; Lee, Lessler, & Stuart, 2010).

The GBM also allows for mediation analysis (Nguyen, Schmid, & Stuart, 2021; Tingley, Yamamoto, Hirose, Keele, & Imai, 2014). The goal of mediation analysis is to determine to what extent the total effect of a variable of interest on an outcome is mediated by an intermediate variable. In our analysis, we consider the role institutional factors play in mediating the relationship between household decision-making power and autonomy in contraceptive use. Mediation analysis is based on the sequential ignorability assumption (K. Imai, Tingley, & Yamamoto, 2013) The assumption comes in two parts (i) it assumes treatment assignment is ignorable given observed covariates (ii) mediator is ignorable given the observed treatment and pre-treatment confounders. While these assumptions are not testable observing balance in the covariates makes them more plausible. Under this assumption the total effect of a treatment on an outcome can be decomposed into a direct effect and an indirect

effect, where the indirect effect is via the mediator. As noted earlier, since institutional factors determine the basis of potentiality for autonomy, it is reasonable to expect that the relationship between autonomous status in household decision-making power and contraceptive use will be mediated by institutional factors. For technical details on this methodology see Nguyen *et al.* (Nguyen et al., 2021).

The framework used in both the causal and the causal mediation analysis (under CIA) can be summarized as follows. Let Y_1 & Y_0 be potential outcome values (lack of autonomy in contraceptive use or the converse) that depends on the exposure of autonomy in household decision-making power $(T = 1)$ or lack thereof $(T = 0)$. Further, say there are two potential mediator values i.e., the household head practises Christianity and respondent lives in Lagos state $(M = 1)$ and household head belongs to other religious groups (i.e., Islam and other religion) and respondent lives in Kano state $(M = 0)$. When exposure values are crossed with potential mediator values the potential outcomes denoted $Y_{(T,M)}$ has four potential states i.e., $Y_{(1,1)}, Y_{(0,0)}, Y_{(1,0)}, Y_{(0,1)}$[1]. The average treatment effect (TE) is given by

$$TE = E(Y_{(1,1)} - Y_{(0,0)})$$

where the expectation is taken over individuals. If we write the above equation as

$$TE = E(Y_{(1,1)} - Y_{(1,0)} + Y_{(1,0)} - Y_{(0,0)})$$

[1] For example, $Y_{(1,0)}$ is the potential outcome when $T = 1$ and $M = 0$

The natural direct effect (NDE) and indirect effect (NIE) are defined as

$$NIE_1 = E(Y_{(1,1)} - Y_{(1,0)})$$

$$NDE_0 = E(Y_{(1,0)} - Y_{(0,0)})$$

Note that when

$$TE = E(Y_{(1,1)} - Y_{(0,1)} + Y_{(0,1)} - Y_{(0,0)})$$

Then

$$NDE_1 = E(Y_{(1,1)} - Y_{(0,1)})$$

$$NIE_0 = E(Y_{(0,1)} - Y_{(0,0)})$$

The main quantity of interest is the NIE_1 i.e., the natural indirect effect in the treatment group. Note NDE_i and NIE_i represents natural direct effect and natural indirect effect in group i, where i is the treatment status.

Results

The descriptive statistics (Table 1) show substantial differences in the contraceptive autonomy, decision-making power, and economic empowerment between women in Kano and Lagos. While 23.1 percent of women in Kano report lacking contraceptive autonomy, only 8.7 percent report lacking autonomy in Lagos. For women in Kano, the lack of autonomy is disproportionately concentrated in the choice not to use contraception (LAC_3). Women in Kano are also less likely to be sole or joint

decision-makers (28 percent) than women in Lagos (76 percent) and are more likely to be non-decision-makers (50.4 percent) than women in Lagos (10.3 percent). Kano consists predominantly of an Islamic population (98.9 percent), while Lagos consists of both large proportions of Christian (64.9 percent) and Islamic (33.9 percent) women. Women in Lagos are more likely to own land, work and earn cash, and have health insurance and savings than women in Kano. The descriptive statistics show that, consistent with the literature, Hausa women live largely in Kano, while Igbo and Yoruba women live largely in the South.

Table 1: Descriptive statistics of dependent and independent variables, disaggregated by city

		Kano	Lagos		
		Mean	Mean	Difference	P-value
Contraceptive autonomy	Lack of contraceptive autonomy	0.23	0.09	0.14	0
	Lack of autonomy in choice to use contraception (non-autonomous use)	0.04	0.04	0	0.62
	Lack of autonomy in choice to not use contraception (non-autonomous non-use)	0.19	0.05	0.15	0
Decision-making power	Sole or joint decision-maker	0.28	0.76	-0.48	0
	Non-decision-maker	0.5	0.1	0.4	0
Religion	Christian	0.01	0.65	-0.64	0
	Islamic	0.99	0.34	0.65	0
	Other	0	0.01	-0.01	0
Marital Status	Currently married	1	0.91	0.09	0
	Currently living with partner	0	0.09	-0.09	0
Polygamous relationship		0.46	0.12	0.34	0
Landowner		0.12	0.26	-0.13	0
Earning status					
	Cash	0.53	0.83	-0.31	0.00
	Cash and kind	0.07	0.02	0.06	0.00
	In-kind	0	0	0	0

	Not paid	0.01	0.03	-0.01	0.04
	Does not work	0.39	0.12	0.26	0.00
Log of years of cohabiting		8.13	8	0.12	0.01
Age in years		30.19	35.4	-5.21	0
Household head		0.010	0.03	-0.03	0
Has health insurance		0.04	0.11	-0.08	0
Has savings		0.4	0.7	-0.3	0
Wealth status	Quintile 1 (poorest)	0.21	0.13	0.07	0
	Quintile 2	0.2	0.21	-0.01	0.55
	Quintile 3	0.19	0.21	-0.02	0.37
	Quintile 4	0.18	0.21	-0.04	0.05
	Quintile 5 (wealthiest)	0.23	0.23	-0.01	0.8
Ethnicity	Hausa	0.84	0.02	0.82	0
	Igbo	0.01	0.19	-0.18	0
	Yoruba	0	0.63	-0.63	0
	Others	0.04	0.17	-0.13	0
Sexual autonomy and contraceptive knowledge	Perceived lack of sexual autonomy	0.42	0.26	0.16	0
	Sexual autonomy	1.01	2.15	-1.14	0
	Contraceptive knowledge	6.61	9.79	-3.18	0
Observations		788	865		

Table 2: Logit regressions: Level of contraceptive autonomy on autonomy in household decision-making power

		Lack of contraceptive autonomy	Lack of contraceptive autonomy	Lack of autonomy in choice to use contraception	Lack of autonomy in choice to not use contraception
Sole or joint decision-maker		-0.37**	-0.27	0.48	-0.51**
		(0.16)	(0.17)	(0.31)	(0.2)
Lagos			-1.24***	0.38	-1.69***
			(0.41)	(0.71)	(0.52)
Religion of household head	Christian		(reference)	(reference)	(reference)
	Islamic		0.08	0.15	0.03
			(0.3)	(0.43)	(0.4)
	Other		3.40***	3.92***	1.88**
			(0.67)	(0.76)	(0.83)
Marital status	Currently married	(reference)	(reference)	(reference)	(reference)
	Currently living with partner	-0.17	-0.09	0.79	-0.45
		(0.42)	(0.44)	(0.6)	(0.63)
Polygamous relationship		0.39**	0.30*	0.42	0.22
		(0.16)	(0.16)	(0.32)	(0.18)
Landowner		0.1	0.16	-0.33	0.32

		(0.19)	(0.2)	(0.35)	(0.23)
Earning status	Cash	(reference)	(reference)	(reference)	(reference)
	Cash and kind	0.39	0.29	0.72	0.09
		(0.31)	(0.32)	(0.53)	(0.37)
	In-kind	0.85	0.74	2.07*	-0.01
		(0.9)	(0.91)	(1.16)	(1.16)
	Not paid	0.11	0.16	0.66	-0.11
		(0.52)	(0.53)	(0.78)	(0.66)
	Does not work	-0.16	-0.22	-0.35	-0.23
		(0.18)	(0.18)	(0.4)	(0.2)
Log of years of cohabiting		-0.03	-0.09	0.59**	-0.22**
		(0.1)	(0.1)	(0.27)	(0.1)
Age in years		<0.001	<0.001	-0.05	0.02
		(0.01)	(0.01)	(0.03)	(0.01)
Has health insurance		0.56**	0.70**	0.19	0.89**
		(0.27)	(0.28)	(0.42)	(0.35)
Has savings		-0.24	-0.23	0.12	-0.34*
		(0.15)	(0.16)	(0.31)	(0.18)
Wealth Quintile	Quintile 1 (poorest)	(reference)	(reference)	(reference)	(reference)
	Quintile 2	0.22	0.32	0.73	0.25
		(0.24)	(0.24)	(0.67)	(0.26)
	Quintile 3	0.58**	0.66***	1.38**	0.50*
		(0.24)	(0.24)	(0.64)	(0.26)

	Quintile 4	0.23	0.31	1.09	0.19
		(0.25)	(0.26)	(0.67)	(0.28)
	Quintile 5 (wealthiest)	0.47*	0.42	2.04***	-0.22
		(0.26)	(0.27)	(0.64)	(0.32)
Ethnicity	Hausa	(reference)	(reference)	(reference)	(reference)
	Igbo	-0.33	0.42	-0.51	0.65
		(0.31)	(0.42)	(0.77)	(0.52)
	Yoruba	-0.67***	0.31	-0.47	0.41
		(0.23)	(0.38)	(0.7)	(0.47)
	Others	0.03	0.43*	-0.3	0.52**
		(0.2)	(0.22)	(0.61)	(0.23)
Household head		0.42	0.6	-0.28	0.97*
		(0.48)	(0.5)	(1.06)	(0.54)
Perceived lack of sexual autonomy		0.11	0.11	0.22	0.07
		(0.09)	(0.09)	(0.18)	(0.1)
Sexual autonomy		-0.13***	-0.12**	-0.02	-0.14**
		(0.05)	(0.05)	(0.09)	(0.06)
Contraceptive knowledge		-0.01	0	0.11**	-0.03
		(0.02)	(0.02)	(0.05)	(0.03)
Observations		1,652	1,652	1,652	1,652

***p<0.01 **p<0.05 ***p<0.1

In Table 2, we report the results from the logistics regression of our contraceptive autonomy variables onto our set of covariates. In column (1), we regress lack of contraceptive autonomy (LAC_1) onto household decision-making power and other covariates, excluding our cultural context and institutional variables (religion and state). These factors are included in column (2). In column (3) and (4), our model is the same as in column (2), except we change our dependent variables to lack of autonomy in the choice to use contraception LAC_2 (column (3)) and lack of autonomy in choice to *not* use contraception LAC_3 (column (4)).

The main results show that those who are joint or sole decision makers in the household are less likely to report lacking autonomy when it comes to contraceptive use (Column (1)). State and religion are also statistically significant predictors of contraceptive autonomy. Households from other religions are statistically significantly more likely to lack contraceptive autonomy (LAC_1) relative to households with Christian household heads (Column (2)). Living in Lagos as opposed to Kano is negatively associated with LAC_1, i.e., women living in Lagos have more contraceptive autonomy than in Kano. However, Column (2) reveals the importance of institutions and context as a potential ameliorating factor in the relationship between contraceptive autonomy and decision-making power. After controlling for state and religion, the result of being a sole or joint decision maker becomes smaller and statistically insignificant. This suggests that institutional environment is important in the relationship between women women's empowerment and contraceptive autonomy. The last two columns show that the negative relationship in column 1 is driven by those whose lack of autonomy results in their non-use of contraception.

Table 3 shows the result for those who are less likely to be involved in household decision-making. Unlike the previous result, those who less likely to be involved in household decision-making are also more likely to report lacking autonomy in contraceptive use. Consistent with the result in Table 2, column 2 show that once state is controlled for the relationship between general autonomy and contraceptive use become statistically insignificant. Lastly columns 3 and 4 show that the positive result between lack of autonomy in general and lack of autonomy is contraceptive use is driven by those whose lack of autonomy results in their non-use of contraception.

Table 3: Logit regressions: Level of contraceptive autonomy on lack of autonomy in household decision-making power

		Lack of contraceptive autonomy	Lack of contraceptive autonomy	Lack of autonomy in choice to use contraception	Lack of autonomy in choice to not use contraception
Non-decision-maker		0.34**	0.22	-0.6	0.37**
		(0.16)	(0.17)	(0.38)	(0.18)
Lagos			-1.25***	0.38	-1.73***
			(0.41)	(0.71)	(0.51)
Religion of household head	Christian		(reference)	(reference)	(reference)
	Islamic		0.09	0.14	0.06
			(0.29)	(0.43)	(0.39)
	Other		3.39***	3.95***	1.86**
			(0.67)	(0.76)	(0.83)
Marital status	Currently married	(reference)	(reference)	(reference)	(reference)
	Currently living with partner	-0.14	-0.07	0.73	-0.43
		(0.43)	(0.44)	(0.6)	(0.63)
Polygamous relationship		0.39**	0.31*	0.41	0.23
		(0.16)	(0.16)	(0.32)	(0.18)

Landowner		0.11	0.17	-0.35	0.34
		(0.19)	(0.2)	(0.35)	(0.23)
Earning status	Cash	(reference)	(reference)	(reference)	(reference)
	Cash and kind	0.41	0.29	0.66	0.07
		(0.31)	(0.32)	(0.53)	(0.37)
	In-kind	0.88	0.76	2.05*	0.03
		(0.91)	(0.91)	(1.16)	(1.17)
	Not paid	0.21	0.23	0.51	0.01
		(0.52)	(0.52)	(0.78)	(0.66)
	Does not work	-0.12	-0.2	-0.4	-0.18
		(0.17)	(0.18)	(0.4)	(0.2)
Log of years of cohabiting		-0.02	-0.08	0.59**	-0.21**
		(0.09)	(0.1)	(0.28)	(0.1)
Age in years		<0.001	<0.001	-0.05	0.01
		(0.01)	(0.01)	(0.03)	(0.01)
Has health insurance		0.54**	0.69**	0.22	0.87**
		(0.27)	(0.28)	(0.42)	(0.35)
Has savings		-0.23	-0.22	0.12	-0.32*
		(0.15)	(0.16)	(0.31)	(0.18)
Wealth Quintile	Quintile 1 (poorest)	(reference)	(reference)	(reference)	(reference)
	Quintile 2	0.23	0.32	0.78	0.26
		(0.24)	(0.24)	(0.67)	(0.26)
	Quintile 3	0.58**	0.66***	1.41**	0.50*
		(0.24)	(0.24)	(0.64)	(0.26)

	Quintile 4	0.23	0.31	1.14*	0.17
		(0.25)	(0.26)	(0.67)	(0.28)
	Quintile 5 (wealthiest)	0.47*	0.41	2.07***	-0.23
		(0.26)	(0.27)	(0.64)	(0.32)
Ethnicity	Hausa	(reference)	(reference)	(reference)	(reference)
	Igbo	-0.37	0.4	-0.5	0.62
		(0.3)	(0.42)	(0.77)	(0.51)
	Yoruba	-0.69***	0.3	-0.46	0.39
		(0.23)	(0.38)	(0.7)	(0.47)
	Others	0.01	0.43*	-0.29	0.51**
		(0.2)	(0.22)	(0.61)	(0.23)
Household head		0.41	0.58	-0.3	0.91*
		(0.48)	(0.5)	(1.06)	(0.54)
Perceived lack of sexual autonomy		0.11	0.12	0.21	0.08
		(0.09)	(0.09)	(0.18)	(0.1)
Sexual autonomy		-0.14***	-0.12**	-0.01	-0.14**
		(0.05)	(0.05)	(0.09)	(0.06)
Contraceptive knowledge		-0.01	0	0.10**	-0.03
		(0.02)	(0.02)	(0.04)	(0.03)
Observations		1,652	1,652	1,652	1,652

***p<0.01 **p<0.05 ***p<0.1

Before presenting the result of the GBM model, Figs 1 and 2 show the balance statistics for the main effects presented in Table 4 (first row of Table 4). Specifically, the GBM model minimized the mean of the standardized difference in means across covariates (the Figures also shows the result when the Kolmogorov-Smirnov statistic is minimized). It is clear that the GBM weights reduce imbalance across the covariates, with the weighted standardized differences in means of all covariates falling below the 0.1 or 0.2 threshold recommended in the literature (Austin, 2009; Stuart et al., 2013). This makes the first part of the sequential ignorability assumption more plausible. Figs S1 and S2 in the supplementary material shows the balance statistics for the total effects (similar to the ones shown here) and the elements of the mediation effects (i.e., $NIE_1, NIE_0, NDE_1 and\ NDE_0$). Similar statistics for the disaggregated analysis (LAC_2 and LAC_3) are shown in the supplementary material (Figs S3-S6).

Fig 1: Balance statistics for sole and joint decision-makers (main effect)

Fig 2: Balance statistics for non-decision makers (main effect)

The results from the mediation analysis for LAC1 (contraceptive use and non-use) are reported in Table 4. For the GBM we use the following parameters, the maximum number of GBM iterations is 10, 000, the shrinkage parameter is set at 0.01 and the level of interactions allowed is 3. The substantive result is consistent with the result from the logit model, although the estimates are smaller in magnitude (suggesting the selection on observables matter). Further, the additional mediation analysis show that while institutional variables mediate the causal relationship for those who have sole or joint household decision-making power, this is not the case for those who lack household decision-making power. Specifically, NDE_1 shows that about 28 percent (-0.02/-0.07) of the total effect of decision-making power on contraceptive autonomy is via the institutional variables. While for those who lack general household autonomy the effect is direct and the effect through the mediators is not statistically

significant. This suggests that the mediators only come into play (to reinforce the direct effect) when women have decision-making power in the household.

Table 4: Household autonomy and autonomy in contraceptive use and non-use (LAC$_1$)

	Sole or joint decision-maker				Non-decision-maker			
	Effect	Standard Error	Confidence Interval Minimum	Confidence Interval Maximum	Effect	Standard Error	Confidence Interval Minimum	Confidence Interval Maximum
Treatment effect	**-0.07**	0.02	-0.11	-0.03	**0.05**	0.03	<0.01	0.1
Natural Direct effect - Control group NDE_0	**-0.05**	0.02	-0.1	-0.01	0.04	0.02	-0.01	0.08
Natural Indirect effect - Treatment group NIE_1	**-0.02**	0.01	-0.02	-0.01	0.01	0.01	-0.01	0.03
Natural Direct effect - Treatment group NDE_1	**-0.06**	0.02	-0.1	-0.01	**0.05**	0.03	<0.01	0.1
Natural Indirect effect - Control group NIE_0	**-0.01**	0.005	-0.02	<-0.01	0.00	0.01	-0.01	0.01

*Note: Emboldened text is statistically significant at a 5% level.

In the supplementary material (Table S1 and S2), we present the results for non-autonomous contraceptive use (LAC$_2$) and non-use (LAC$_3$). The result show similar pattern to the logit model, with non-autonomous non-use (LAC$_3$) being significant and non-autonomous use (LAC$_2$) being statistically insignificant (after controlling for selection on observables). Furthermore, for the mediation analysis, the mediation quantity of interest NIE_1 is only significant for sole/joint decision makers in the case of non-autonomous non-use.

Discussion

This paper finds evidence of a relationship between household decision-making power and contraceptive autonomy, controlling for selection on observables. Household decision-making power may influence contraceptive autonomy through a myriad of avenues. Firstly, decision-making power may reflect financial independence and empowerment, which has been associated with the acceptability of discussing fertility choices and family planning with a partner (Hogan, Berhanu, &

Hailemariam, 1999). Economic decision-making power and education have also been associated with lower levels of unmet contraception needs, likely through better access to health facilities (Ali & Okud, 2013). Decision-making power improves knowledge of contraception (Hogan et al., 1999), which in turn is related to the decision to use contraception (Hailemariam & Haddis, 2011; Wulifan, Brenner, Jahn, & De Allegri, 2015).

However, this relationship between household decision-making power and contraceptive autonomy has to some extent been established in the literature. The unique contribution of this paper is the finding that show the overpowering role of institutions in mediating the potential impact that decision-making power can play in improving women's contraceptive autonomy. We firstly explore the relationship using logit regressions, and find that although there is a significant relationship between decision-making power and a lack of overall contraceptive autonomy (LAC_1), this relationship disappears when we control for state and religion. This result is found when we focus on sole or joint decision makers where there is a negative relationship with LAC_1, and non-decision-makers, where there is a positive relationship with LAC_1.

Secondly, novel mediation analysis is used to test whether the role of institutional variables as an ameliorating factor holds under a more rigorous empirical application. We found that institutions matter only for women who have sole or joint decision-making power. In fact, 28 percent of the total effect of sole or joint decision-making power on contraceptive autonomy is via the role of the women's state and religion. The same is not true for non-decision makers. Being a non-decision-maker directly affects women's contraceptive autonomy, regardless of their state or religion.

Our finding supports the theory that institutions play an important role in establishing the boundaries of women's autonomy, and therefore, their contraceptive autonomy.

The lack of the mediatory contribution of institutions and religion to contraceptive autonomy for non-decision-makers may highlight the potential severity of household-level disempowerment of women. Regardless of their community's adoption of specific norms and attitudes towards contraception, women who lack decision-making power in the household lack choice in various aspects of their daily existence. Autonomy in one area of life often manifests in other areas of life.

There have been many individual-level interventions to improve access to family planning services to reach women alone (F. OlaOlorun, 2010). For instance, one study in South Africa tested the feasibility of providing women with contraception and counselling at hairdressers, as it is considered a safe, community-based space which is predominantly frequented by women. Offering contraception in this space would allow women to make decisions regarding their sexual- and reproductive health independently from their partners (Bassett et al., 2018). Evidence in Zambia points to a similar strategy in improving contraceptive access. Ashraf *et al.* in a Randomised Controlled Trial tested the difference in contraceptive access when women were offered a voucher to access contraception by themselves, or when access required their partners approval. The authors found that involving spouses in family planning visits resulted in a significant drop in contraceptive access. However, the authors do note that two years after the experiment, there is a significant emotional cost to women who had made their contraception choices in isolation (Ashraf, Field, & Lee, 2014).

While we are unable to assess the healthcare systems and access to concealable contraception among our sample of women, we do find that there is a portion of women who are choice-constrained (both in their household decision-making and in their choice not to use of contraception), and this is

do you mean religious affiliation here?

independent of their state and belief. Improving physical access to concealable contraception received in privacy may not be a strong enough stand-alone policy to address this level of disempowerment.

Conclusion

Institutions, culture, and context can play a key role in empowering women, emboldening their decision-making power, and enhancing their contraceptive autonomy. The paper quantifies the importance of engraining gender equality into discussions around sexual and reproductive health, even more so in contexts where specific values suppress contraceptive and reproductive autonomy. Our analysis is not without limitations, importantly we note that the relationships identified are in terms of correlation between the variables of interest and may not have causal interpretation since we are unable to control for unobserved factors.

word choice?

;

flesh out discussion and conclusion

References

Adebowale, A. S. (2019). Ethnic disparities in fertility and its determinants in Nigeria. *Fertility research and practice, 5*(1), 1-16.

Ali, A. A. A., & Okud, A. (2013). Factors affecting unmet need for family planning in Eastern Sudan. *BMC public health, 13*(1), 1-5.

Ashraf, N., Field, E., & Lee, J. (2014). Household bargaining and excess fertility: an experimental study in Zambia. *American Economic Review, 104*(7), 2210-2237.

Bassett, I. V., Xu, A., Govere, S., Thulare, H., Frank, S. C., Psaros, C., & Parker, R. A. (2018). Contraception and PrEP in South African hair salons: Owner, stylist, and client views. *Journal of Acquired Immune Deficiency Syndromes (1999), 79*(2), e78.

Bongaarts, J. (2017). The effect of contraception on fertility: Is sub-Saharan Africa different? *Demographic Research, 37*.

Brown, S. S., & Eisenberg, L. (1995). Unintended pregnancy and the well-being of children and families. *JAMA, 274*(17), 1332-1332.

Centre for Research, E. R. a. D. C., Bayero University Kano (BUK), & The Bill & Melinda Gates Institute for Population and Reproductive Health at The Johns Hopkins Bloomberg School of Public Health. (2020). *Performance Monitoring for Action (PMA) Household and Female Survey Phase 1, PMA/Nigeria-HQFQ-P1 (Kano & Lagos).*

Chirkov, V. (2017). Culture and autonomy. In A. T. Church (Ed.), *The Praeger Handbook of Personality Across Cultures [3 volumes]*: ABC-CLIO.

Chirkov, V. I., Ryan, R., & Sheldon, K. M. (2010). *Human autonomy in cross-cultural context: Perspectives on the psychology of agency, freedom, and well-being* (Vol. 1): Springer Science & Business Media.

Choi, B. Y., Wang, C.-P., Michalek, J., & Gelfond, J. (2019). Power comparison for propensity score methods. *Computational Statistics, 34*(2), 743-761.

Collier, Z. K., Leite, W. L., & Zhang, H. (2021). Estimating propensity scores using neural networks and traditional methods: a comparative simulation study. *Communications in Statistics-Simulation and Computation*, 1-16.

Diamond, A., & Sekhon, J. S. (2013). Genetic matching for estimating causal effects: A general multivariate matching method for achieving balance in observational studies. *Review of Economics and Statistics, 95*(3), 932-945.

Do, M., & Kurimoto, N. (2012). Women's empowerment and choice of contraceptive methods in selected African countries. *International perspectives on sexual and reproductive health*, 23-33.

Elaigwu, J. I., & Galadima, H. (2003). The shadow of Sharia over Nigerian federalism. *Publius: The Journal of Federalism, 33*(3), 123-144.

Fadiran, D. O., Sarr, M., & Fedderke, J. W. (2017). Measurement and Analysis of the Evolution of Institutions in Nigeria. *Lagos, Nigeria: Islamic Publications Bureau.*

Friedman, J. H. (2001). Greedy function approximation: a gradient boosting machine. *Annals of statistics*, 1189-1232.

Hailemariam, A., & Haddis, F. (2011). Factors affecting unmet need for family planning in southern nations, nationalities and peoples region, Ethiopia. *Ethiopian journal of health sciences, 21*(2), 77-90.

Hogan, D. P., Berhanu, B., & Hailemariam, A. (1999). Household organization, women's autonomy, and contraceptive behavior in southern Ethiopia. *Studies in family planning, 30*(4), 302-314.

Imai, K., & Ratkovic, M. (2014). Covariate balancing propensity score. *Journal of the Royal Statistical Society: Series B (Statistical Methodology), 76*(1), 243-263.

Imai, K., Tingley, D., & Yamamoto, T. (2013). Experimental designs for identifying causal mechanisms. *Journal of the Royal Statistical Society: Series A (Statistics in Society), 176*(1), 5-51.

Kendhammer, B. (2013). The Sharia controversy in Northern Nigeria and the politics of Islamic law in new and uncertain democracies. *Comparative Politics, 45*(3), 291-311.

Lee, B. K., Lessler, J., & Stuart, E. A. (2010). Improving propensity score weighting using machine learning. *Statistics in medicine, 29*(3), 337-346.

McCaffrey, D. F., Ridgeway, G., & Morral, A. R. (2004). Propensity score estimation with boosted regression for evaluating causal effects in observational studies. *Psychological methods, 9*(4), 403.

Mutumba, M., Wekesa, E., & Stephenson, R. (2018). Community influences on modern contraceptive use among young women in low and middle-income countries: a cross-sectional multi-country analysis. *BMC public health, 18*(1), 1-9.

National Population Commission. (2013). *Nigeria demographic and health survey 2013*. Retrieved from

National Population Commission. (2015). *Nigerian Population Census.*

Nguyen, T. Q., Schmid, I., & Stuart, E. A. (2021). Clarifying causal mediation analysis for the applied researcher: Defining effects based on what we want to learn. *Psychological methods, 26*(2), 255.

Nkoka, O., Lee, D., Chuang, K.-Y., & Chuang, Y.-C. (2021). Multilevel analysis of the role of women's empowerment on use of contraceptive methods among married Cambodian women: evidence from demographic health surveys between 2005 and 2014. *BMC Women's Health, 21*(1), 1-13.

North, D. C. (1991). Institutions. *Journal of economic perspectives, 5*(1), 97-112.

Nwadialor, K. L. (2013). Christian Missionaries and Civilization in Southern Nigeria, 1841-1960: Implications for Contemporary Christians. *UJAH: Unizik Journal of Arts and Humanities, 14*(2), 173-193.

Ojo, O. (2008). The Organization of the Atlantic Slave Trade in Yorubaland, ca. 1777 to ca. 1856. *The International journal of African historical studies, 41*(1), 77-100.

OlaOlorun, F. (2010). Advancing family planning research in Africa. *African journal of reproductive health, 14*(4).

OlaOlorun, F. M., & Hindin, M. J. (2014). Having a say matters: influence of decision-making power on contraceptive use among Nigerian women ages 35–49 years. *PloS one, 9*(6), e98702.

Ostien, P., & Dekker, A. (2010). Sharia and national law in Nigeria. *Sharia incorporated: A comparative overview of the legal systems of twelve Muslim countries in past and present*, 553-612.

Park, M. S.-A., & Chirkov, V. (2020). Culture, Self, and Autonomy. *Frontiers in Psychology, 11*, 736.

Patrikar, S., Basannar, D., & Sharma, M. S. (2014). Women empowerment and use of contraception. *Medical journal armed forces India, 70*(3), 253-256.

Rettig, E. M., Fick, S. E., & Hijmans, R. J. (2020). The Female Empowerment Index (FEMI): spatial and temporal variation in women's empowerment in Nigeria. *Heliyon, 6*(5), e03829.

Ridgeway, G., McCaffrey, D., Morral, A., Cefalu, M., Burgette, L., Pane, J., & Griffin, B. A. (2021). Toolkit for Weighting and Analysis of Nonequivalent Groups: A guide to the twang package. *vignette, July, 26*.

Rosenbaum, P. R., & Rubin, D. B. (1983). The central role of the propensity score in observational studies for causal effects. *Biometrika, 70*(1), 41-55.

The World Bank. (2022a). Fertility rates, total (births) per woman. Retrieved from https://data.worldbank.org/indicator/SP.DYN.TFRT.IN?locations=NG

The World Bank. (2022b). Population, total - Nigeria. Retrieved from https://data.worldbank.org/indicator/SP.POP.TOTL?locations=NG

Tingley, D., Yamamoto, T., Hirose, K., Keele, L., & Imai, K. (2014). Mediation: R package for causal mediation analysis.

Westerlund, D., & Svanberg, I. (1999). *Islam outside the Arab world*: Psychology Press.

Woldemicael, G. (2009). Women's autonomy and reproductive preferences in Eritrea. *Journal of biosocial science, 41*(2), 161-181.

Wulifan, J. K., Brenner, S., Jahn, A., & De Allegri, M. (2015). A scoping review on determinants of unmet need for family planning among women of reproductive age in low and middle income countries. *BMC Women's Health, 16*(1), 1-15.

Yaya, S., Uthman, O. A., Ekholuenetale, M., & Bishwajit, G. (2018). Women empowerment as an enabling factor of contraceptive use in sub-Saharan Africa: a multilevel analysis of cross-sectional surveys of 32 countries. *Reproductive health, 15*(1), 1-12.

Zhao, Z. (2008). Sensitivity of propensity score methods to the specifications. *Economics Letters, 98*(3), 309-319.

Supporting Information

Table S1: Household autonomy and autonomy in contraceptive use (LAC_2)
Table S2: Household autonomy and autonomy in contraceptive non-use (LAC_3)
Fig S1: Balance statistics for non-decision makers (mediation effect)
Fig S2: Balance statistics for sole and joint decision makers (mediation effect)
Fig S3: Balance statistics for non-decision makers (mediation effect) (LAC_2)
Fig S4: Balance statistics for sole or joint makers (mediation effect) (LAC_2)
Fig S5: Balance statistics for non-decision makers (mediation effect) (LAC_3)
Fig S6: Balance statistics for sole or joint makers (mediation effect) (LAC_3)

Figure

Click here to access/download;Figure;Figure 1.jpg

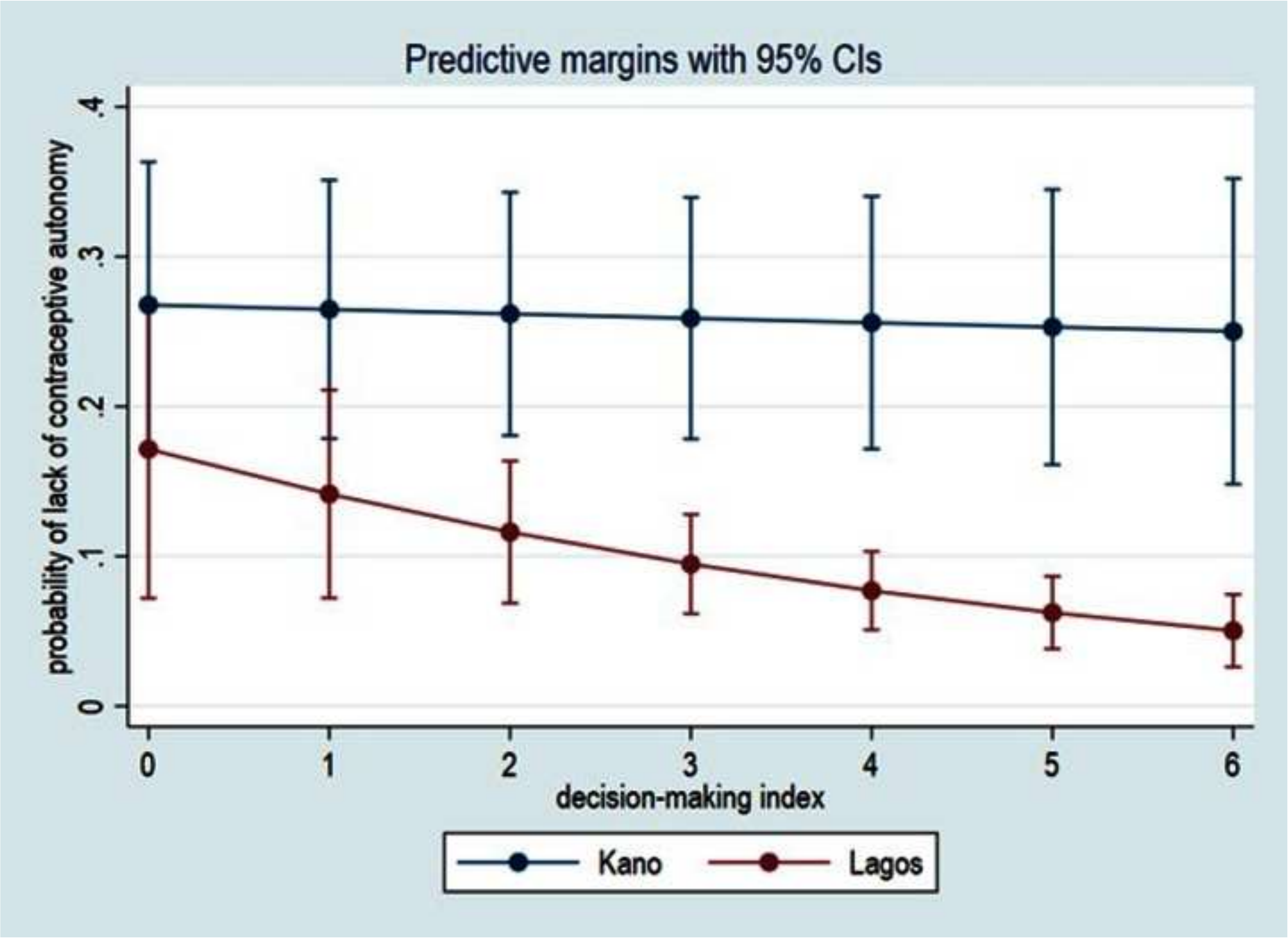

Manuscript

Title: 'Institutions matter: The role of institutions in the relationship between decision-making power and contraceptive autonomy'

Short title: Institutions, decision-making power and contraceptive autonomy.

Corresponding author: Laura Rossouw, School of Economics and Finance, University of the Witwatersrand, Johannesburg, South Africa. http://orcid.org/0000-0001-7697-9567. Email: laura.rossouw@wits.ac.za; Telephone:+27 (0)11 717 9999

Adeola Oyenubi, School of Economics and Finance, University of the Witwatersrand, Johannesburg, South Africa. http://orcid.org/0000-0001-6068-1723

Abstract:

Progress in the development of modern contraception is recognized as one of the 10 great public health achievements of the 20th century. Several academic papers have shown that the distribution of household decision-making power has a positive influence on women's contraceptive autonomy. This paper considers the role of the social or institutional environment (as captured by ~~religion and~~ place of residence) in ameliorating or contributing to this relationship. Our study focuses on the context of Nigeria, a country with diverse gender norms and religious practices, often determined by geographic location. For example, Western common law is more prevalent in the Southern Nigeria, while Sharia Law is largely practiced in the North of the country. The analysis uses the Performance and Monitoring for Action (PMA) dataset (2019-2020), and analysis is done using logistic regressions. ~~we control for selection on observables using the Gradient boosted model.~~ We find evidence that the relationship between household decision-making power and contraceptive autonomy is mediated by institutions (city ~~and religion~~). More specifically, we show that in Lagos – a state characterised by the increasing empowerment of women – women have more contraceptive autonomy as they gain decision-making power in the household. The opposite is true in Kano - household decision-making power has no bearing on contraceptive autonomy. ~~, but only for women with some decision-making power in their household. Among women with no decision-making power in the household, institutions do not play a role in their contraceptive autonomy. This finding highlights the potential severity of household level disempowerment of women.~~

Keywords: Decision-making power; Autonomy; Contraception; Empowerment; Gender

Word count: 5904 words

Introduction

The fertility rate in Nigeria has been steadily declining, from an average of 6.4 births per woman in 1960 to 5.3 in 2019. Despite the incremental decline, this fertility rate is almost double the average in other lower middle income countries (The World Bank, 2022a). However, with a population of over 206 million (The World Bank, 2022b), Nigeria is a large and diverse country with 36 states, several ethnic groups and different socio-cultural identities (Adebowale, 2019), and average reproductive trends may misconstrue state-level tendencies. Most notably, Nigerian fertility rates are reportedly higher in the Northern states than the Southern states (National Population Commission, 2013). A key predictor of fertility includes access to modern contraception (Bongaarts, 2017). In 2013, access to contraception by women in Nigeria was still heavily skewed to the Southern States at 44 percent, compared to 12 percent in the Northern States (Rettig, Fick, & Hijmans, 2020).

Access to modern contraceptive methods is a function of several structural, cultural, and socio-economic factors, including women's empowerment and household bargaining power (Do & Kurimoto, 2012; Nkoka, Lee, Chuang, & Chuang, 2021; F. M. OlaOlorun & Hindin, 2014; Patrikar, Basannar, & Sharma, 2014; Yaya, Uthman, Ekholuenetale, & Bishwajit, 2018). Contraceptive knowledge, access and use are shaped by personal factors (like autonomy, attitudes and feelings about contraception), community influences (Mutumba, Wekesa, & Stephenson, 2018), and the surrounding socioeconomic and cultural environment (i.e. informal institutions) (Brown & Eisenberg, 1995). The role of the cultural environment and informal institutions in shaping women's empowerment and

household bargaining power, and therefore, access to modern contraception is an area that has not been evaluated extensively.

In this article, we investigate the relationship between informal institutions in Nigeria (captured by the respondent's city and religion) and contraceptive autonomy, as well as the relationship between household decision-making power and contraceptive autonomy. Finally we explore how institutions and household decision-making power interact to affect contraceptive autonomy. ~~through the impact of these institutions on women's household decision-making power.~~ Contraceptive autonomy refers to the ability of women to access their contraception of choice, but also includes the choice to opt out of using any contraception.

The research question will be evaluated within an institutional theory conceptual framework, which posits that institutions shape individuals' behaviours and interactions within a society. According to North, institutions are 'humanly devised constraints that structure human interactions. They are made up of formal constraints (rules, laws, constitutions), informal constraints (norms of behaviour, convention, and self-imposed codes of conduct), and their enforcement characteristics' (North, 1991).

In our analysis, institutions are defined as social norms and culture as determined by respondents' geo-location. In Nigeria, women's geographical location is likely to determine their ethnic group, lifestyle and often religion (Adebowale, 2019). We propose that institutions like beliefs and community norms play an important role in shaping attitudes and practices related to health and contraception choice, while simultaneously shaping household decision-making power and internal family dynamics.

Institutions may influence household decision-making power and health choices through a myriad of pathways. They may prescribe specific gender role for men and women, which could affect decision-making and the distribution of authority (Sundararajan et al., 2019). Institutions often offer moral and ethical guidance, shaping perceptions about right and wrong which may shape household decision-making within a household (Shariff, 2015). Institutions may also offer household access to communities and social networks, where peer effects in turn might affect decisions around education, health and social activities (Kandpal & Baylis, 2019). Local customs as institutions also often have a direct influence on contraceptive choice and autonomy, discouraging the use of contraception or limit the choice of acceptable contraception types (Hill, Siwatu, & Robinson, 2014; Pinter et al., 2016; Sundararajan et al., 2019).

The expectation that institutional environments or context can influence the relationship between women's empowerment and contraceptive autonomy is derived from the field of psychology (V. Chirkov, 2017; V. I. Chirkov, Ryan, & Sheldon, 2010; Park & Chirkov, 2020). Specifically, the literature on the relationship between culture, self, and autonomy suggests that culture (or context) plays a key role in determining the potential for autonomy (V. Chirkov, 2017). Culture influences the definition of self, and by doing so sets the boundaries for a socially acceptable level of self-determination or autonomy (Park & Chirkov, 2020). The implication is that culture as a context is integral to one's construction of self and therefore autonomy. Therefore, if we accept the premise that women empowerment or autonomy influences the choice to use or not use contraceptives, and empowerment is context or culture specific, then one should expect that context will mediate the relationship between empowerment and contraceptive use. If institutions determine the boundaries of general autonomy and decision-making power, we expect that institutions will affect contraceptive autonomy.

~~In our analysis, institutions are defined as social norms, culture, and religion as determined by respondents' geo-location.~~ In ~~Nigeria, women's geographical location is likely to determine their ethnic group, lifestyle and often religion (Adebowale, 2019).~~ Our analysis is restricted to two Nigerian cities, namely Kano and Lagos. Kano is situated in northern Nigeria where Sharia Law is largely practiced. Lagos is situated in southern Nigeria where western common law is more prevalent (Elaigwu & Galadima, 2003; Kendhammer, 2013; Ostien & Dekker, 2010). The Hausa/Fulani ethnic groups often reside in the North of the country, while the Igbo and Yoruba tribes reside in the South (National Population Commission, 2015). In their national and subnational evaluation of female empowerment in Nigeria, Rettig *et al.* find that while female empowerment increased overall in Nigeria since the 1990s, this is largely driven by improvements in the South, with the Northern Nigerian states remaining relatively unchanged.

Differences in institutions between northern and southern Nigeria can arguably be dated back to pre-colonial days. Prior to the arrival of the British, Arabic traders ventured into the northern Nigeria (Fadiran, Sarr, & Fedderke, 2017). Consequently, Hausa people occupying northern Nigeria have a cultural heritage predominated by Arabic influence. The political system, for example, was based on Islamic law (Fadiran et al., 2017). Western influence and religion is more strongly represented in heritage and the way of life in Southern Nigeria. For example, transatlantic slave trade in Southern Nigeria pre-dated 1790 (Ojo, 2008) while western influence in form of Christian missionaries in southern Nigeria dates back to 1841 (Nwadialor, 2013). This difference in history affects present reality and in part explains differences in institutional environments in present day Nigeria. The analysis of these two cities situated in different states allows us to investigate the potential role of institutions and social norms in the relationship between household decision-making power and contraceptive autonomy.

Methods

Data

For this analysis, we used the Performance Monitoring for Action Nigeria Phase 1 Household and Female Survey Dataset, which focused on contraception, reproductive health, and family planning. The specific Nigerian dataset was chosen as it also explores women's autonomy. The data was sampled using a stratified cluster design with urban-rural strata, based on the National Population Commission's master sampling frame. Data was collected during December 2019 and January 2020. The final sample collected consists of 1122 women in Kano and 1469 women in Lagos (Centre for Research, Bayero University Kano (BUK), & The Bill & Melinda Gates Institute for Population and Reproductive Health at The Johns Hopkins Bloomberg School of Public Health, 2020). We restricted the sample to women who were married or living in a cohabiting relationship, so as do gauge women's contraceptive autonomy within the context of a sexually active relationship and household. As a result, we have a sample of 788 and 865 in Kano and Lagos respectively.

Variables

Dependent variable: Contraceptive autonomy

Our definition of contraceptive autonomy coincides with the modern perspective on reproductive decision-making. Woldemicael writes that the definition of women's autonomy in reproductive decisions shifted after the 1994 International Conference on Population and Development, from joint

or interdependent decision-making with a spouse during the nineties, to emphasizing the role of woman's individual choice (Woldemicael, 2009). This follows the argument of Senderowicz (2020), who noted that non-autonomous use of contraception is also a negative outcome when it comes to autonomy and contraceptive use. By exploring contraceptive autonomy, rather than contraceptive use, we are able to capture the nuanced and dynamic nature of contraceptive choice.

Contraceptive autonomy was captured using the questions 'Would you say that using contraception is mainly your decision' and 'Would you say that not using contraception is mainly your decision'. For both of these questions, respondents could answer that the decision was either a) mainly theirs, b) the decision of their husband/partner, c) a joint decision or d) somebody else made the decision. Using this information, we created ~~three variables of contraceptive autonomy was created~~a ~~:~~

- ~~Lack of contraceptive autonomy (LAC_1): A~~ binary variable equal to one if the respondent is not involved with their contraceptive choices. Either their husband/partner or somebody else made the decision for their contraceptive use or non-use.
- ~~Lack of autonomy in choice to use contraception (LAC_2): A binary variable equal to one if the respondent is not the main decision-maker with regards to using contraception. Either their husband/partner or somebody else made the decision for their contraceptive use.~~
- ~~Lack of autonomy in choice to not use contraception (LAC_3): A binary variable equal to one if the respondent is not the main decision-maker with regards to not using contraception. Either their husband/partner or somebody else made the decision for their contraceptive non-use.~~

~~We include the final two outcome variables in our analysis to test for the possibility that the factors related to non-autonomous use may be different from non-autonomous non-use of contraception.~~

Formatted: Justified

Formatted: Normal, No bullets or numbering

Formatted: Normal, Indent: Left: 0"

Independent variable of interest: Household decision-making power

Household decision-making power was established using a set of six questions. Respondents were asked whom in the household makes decisions around (1) large household purchases, (2) household purchases for daily needs, (3) medical treatment for the respondent, (4) buying clothes for the respondent, (5) the spending of the respondent's earnings, or (6) how the respondent's partner's earnings are spent. For each of these questions, respondents could answer that they either a) made the decision themselves, b) their husband or partner made the decision, c) they made the decision with their partner jointly, or d) someone else decided.

Using this data, we create a decision-making index that counts the number of instances where the respondent is involved in decision making (either solely or jointly). The index ranges from 1 to 6, with a higher value indicating more decision-making power.

~~Using this data, two decision-making categories were created:~~

- ~~Sole or joint decision-maker. A binary variable equal to one if respondents were involved in decision-making (either solely or jointly) in at least three of the six questions listed above, and zero if not.~~
- ~~Non-decision-maker: A binary variable equal to one if the respondent answered that their husband or partner, or someone else made their decisions in at least three of the six questions listed above, and zero if not.~~

Covariates

Our choice of covariates is informed by our institutional theory conceptual framework. We control for several covariates that explain contraceptive autonomy, including marital status ('Currently married', 'Currently living with man') and whether it is polygamous, duration of co-habiting, age,

possession of health insurance, ethnicity (Hausa, Igbo, Yoruba, and others), and whether the respondent is the head of household. The respondent's economic and financial position is captured by including a dummy variable equal to one if the respondent has savings, land ownership, earning status (are they renumerated in 'Cash', 'Cash and kind', 'In-kind', 'Not paid' and 'Not working') and the respondents household wealth quintile.

Our ~~first~~ institutional variable is state, which is a binary variable equal to one if the respondent resides in Lagos and zero if they reside in Kano. ~~Our second institutional variable~~In addition to state, we also include a potential second ~~is~~ institutional variable - a categorical religion variable (Christian, Islamic, Other).

Lastly we control for a variable that captures knowledge of contraception created as a summary index ranging from zero to 16, with a value of one every time a respondent answered that they have heard of the following contraceptive methods: 'female sterilization', 'male sterilization', 'implants', 'IUD', 'injectables', 'birth control pills', 'emergency contraception', 'male condoms', 'female condoms', 'diaphragm', 'foam or jelly as a contraceptive method', 'standard days method or cycle beads', 'LAM', 'rhythm', 'withdrawal' and 'others'.

~~We also control for variables that capture autonomy relating to sexual behaviour in general.~~

- ~~Perceived lack of sexual autonomy: A summary index ranging from zero to four, with a value of one every time a respondent answered yes to the following 'If I refuse sex with my husband/partner, he may stop supporting me', 'If I refuse sex with my husband/partner, he~~

may force me to have sex', 'If I refuse sex with my husband/partner, he may physically hurt me', 'If I show my partner that I want to have sex, he may consider me promiscuous'.

- Sexual autonomy: A summary index ranging from zero to four, with a value of one every time a respondent answered yes to the following 'I am confident I can tell my husband/partner when I want to have sex', 'I am able to decide when to have sex', 'If I do not want to have sex, I can tell my husband/partner', 'If I do not want to have sex, I am capable of avoiding it'.
- Knowledge of contraception: A summary index ranging from zero to 16, with a value of one every time a respondent answered that they have heard of the following contraceptive methods: 'female sterilization', 'male sterilization', 'implants', 'IUD', 'injectables', 'birth control pills', 'emergency contraception', 'male condoms', 'female condoms', 'diaphragm', 'foam or jelly as a contraceptive method', 'standard days method or cycle beads', 'LAM', 'rhythm', 'withdrawal' and 'others'.

Data Analyses

Our analysis uses logistic regression to estimate the relationship between household decision-making power and the level of autonomy in contraceptive use. We account for the role of institution by interacting state with decision-making power to see if the relationship between the two variables depends on institutional context as measured by state. The premise of this analysis is that the relationship between culture, self, and autonomy suggests that culture (or context) plays a key role in determining the potential for autonomy (Chirkov, 2017). Culture influences the definition of self and by doing so, sets the boundaries for what socially acceptable level of self-determination or autonomy (Park & Chirkov, 2020). This suggests that differences in cultural contexts between Kkano and Lagos may influence the relationship between autonomy and contraceptive use. Stata 16 is used to conduct the analysis.

Our analysis consists of two parts. Firstly, we use logistic regression to estimate the relationship between household decision-making power and the level of autonomy in contraceptive use. However, since this approach does not account for differences in distribution of covariates between those who have autonomy in household decision-making and those who do not, we consider a model that accounts for the difference in characteristics. We therefore consider an approach that has causal interpretation under the Conditional Independence Assumption (CIA) (Rosenbaum & Rubin, 1983). However, we note that causal interpretation is only valid under the CIA, since we cannot control for unobserved covariates, this approach can be interpreted as relationship between the variables of interest while controlling for selection based on observed covariates. This approach utilizes the propensity score, or the probability of having general autonomy given observed covariates. The scores are then used to achieve balance in the distribution of covariates (by weighting or matching) so that the resulting estimate is not biased by the difference in distribution of covariates across groups.

A propensity score model specifies the relationship between a set of pre-treatment covariates and a treatment of interest, which in our analysis is household decision-making power. While in general any model relating a set of covariates to group assignment can be used to estimate the propensity scores (e.g., logistic regression), it has been found that misspecification of the propensity score model can lead to bias in the resulting effect estimate (Kosuke Imai & Ratkovic, 2014; Zhao, 2008). To solve this problem, researchers have started using non-parametric, data-driven optimization algorithms to estimate propensity scores (Collier, Leite, & Zhang, 2021; Diamond & Sekhon, 2013; McCaffrey, Ridgeway, & Morral, 2004).

One such method is the Gradient boosted model (GBM) approach (Friedman, 2001). GBM is a machine learning approach that can be used to fit a nonlinear surface and predict group assignment

while reducing bias that may arise from misspecification of the propensity score model. This is because GBM is a non-parametric approach that allows flexible, non-linear relationships in estimating propensity scores. The general idea is to optimize balance in the distribution of covariates across groups by selecting the iteration of the GBM that achieves the best balance (Ridgeway et al., 2021). The main advantage of this approach is that it captures non-linear effects and interactions terms without prior knowledge of the model. By automatically considering higher order terms this approach makes CIA more plausible. GBM has been shown to be less biased than parametric models (Choi, Wang, Michalek, & Gelfond, 2019; Lee, Lessler, & Stuart, 2010).

The GBM also allows for mediation analysis (Nguyen, Schmid, & Stuart, 2021; Tingley, Yamamoto, Hirose, Keele, & Imai, 2014). The goal of mediation analysis is to determine to what extent the total effect of a variable of interest on an outcome is mediated by an intermediate variable. In our analysis, we consider the role institutional factors play in mediating the relationship between household decision-making power and autonomy in contraceptive use. Mediation analysis is based on the sequential ignorability assumption (K. Imai, Tingley, & Yamamoto, 2013) The assumption comes in two parts (i) it assumes treatment assignment is ignorable given observed covariates (ii) mediator is ignorable given the observed treatment and pre-treatment confounders. While these assumptions are not testable observing balance in the covariates makes them more plausible. Under this assumption the total effect of a treatment on an outcome can be decomposed into a direct effect and an indirect effect, where the indirect effect is via the mediator. As noted earlier, since institutional factors determine the basis of potentiality for autonomy, it is reasonable to expect that the relationship between autonomous status in household decision-making power and contraceptive use will be mediated by institutional factors. For technical details on this methodology see Nguyen *et al.* (Nguyen et al., 2021).

The framework used in both the causal and the causal mediation analysis (under CIA) can be summarized as follows. Let Y_1 & Y_0 be potential outcome values (lack of autonomy in contraceptive use or the converse) that depends on the exposure of autonomy in household decision-making power $(T = 1)$ or lack thereof $(T = 0)$. Further, say there are two potential mediator values i.e., the household head practises Christianity and respondent lives in Lagos state $(M = 1)$ and household head belongs to other religious groups (i.e., Islam and other religion) and respondent lives in Kano state $(M = 0)$. When exposure values are crossed with potential mediator values the potential outcomes denoted $Y_{(T,M)}$ has four potential states i.e., $Y_{(1,1)}, Y_{(0,0)}, Y_{(1,0)}, Y_{(0,1)}$[1]. The average treatment effect (TE) is given by

$$TE = E(Y_{(1,1)} - Y_{(0,0)})$$

where the expectation is taken over individuals. If we write the above equation as

$$TE = E(Y_{(1,1)} - Y_{(1,0)} + Y_{(1,0)} - Y_{(0,0)})$$

The natural direct effect (NDE) and indirect effect (NIE) are defined as

$$NIE_1 = E(Y_{(1,1)} - Y_{(1,0)})$$

$$NDE_0 = E(Y_{(1,0)} - Y_{(0,0)})$$

[1] For example, $Y_{(1,0)}$ is the potential outcome when $T = 1$ and $M = 0$

Note that when

$$TE = E(Y_{(1,1)} - Y_{(0,1)} + Y_{(0,1)} - Y_{(0,0)})$$

Then

$$NDE_1 = E(Y_{(1,1)} - Y_{(0,1)})$$

$$NIE_0 = E(Y_{(0,1)} - Y_{(0,0)})$$

The main quantity of interest is the NIE_1 i.e., the natural indirect effect in the treatment group. Note NDE_i and NIE_i represents natural direct effect and natural indirect effect in group i, where i is the treatment status.

Results

The descriptive statistics (Table 1) show substantial differences in the contraceptive autonomy, decision-making power, and economic empowerment between women in Kano and Lagos. While 23.1 percent of women in Kano report lacking contraceptive autonomy, only 8.7 percent report lacking autonomy in Lagos. Further average autonomy index in Lagos is double that of Kano state (4.3 versus 2.3). For women in Kano, the lack of autonomy is disproportionately concentrated in the choice not to use contraception (LAC_3). Women in Kano are also less likely to be sole or joint decision-makers (28 percent) than women in Lagos (76 percent) and are more likely to be non-decision-makers (50.4 percent) than women in Lagos (10.3 percent). Kano consists predominantly of an Islamic population (98.9 percent), while Lagos consists of both large proportion of Christian (64.9 percent) and Islamic (33.9 percent) women. Women in Lagos are more likely to own land, work and earn cash, have health

insurance and savings than women in Kano. The descriptive statistics show that, consistent with the literature, Hausa women live largely in Kano, while Igbo and Yoruba women live largely in the South.

Table 1: Descriptive statistics of dependent and independent variables, disaggregated by city

		Kano	Lagos		
		Mean	Mean	Difference	P-value
No cContraceptive autonomy	Lack of contraceptive autonomy	0.23	0.09	0.14	0
Decision-making power index		2.261	4.284	-2.023	0
	Lack of autonomy in choice to use contraception (non-autonomous use)	0.04	0.04	0	0.62
	Lack of autonomy in choice to not use contraception (non-autonomous non-use)	0.19	0.05	0.15	0
Decision-making power	Sole or joint decision-maker	0.28	0.76	-0.48	0
	Non-decision-maker	0.5	0.1	0.4	0
Religion	Christian	0.01	0.65	-0.64	0
	Islamic	0.99	0.34	0.65	0
	Other	0	0.01	-0.01	0
Marital Status	Currently married	1	0.91	0.09	0
	Currently living with partner	0	0.09	-0.09	0
Polygamous relationship		0.46	0.12	0.34	0
Landowner		0.12	0.26	-0.13	0
Earning status	Cash	0.53	0.83	-0.31	0.00
	Cash and kind	0.07	0.02	0.06	0.00
	In-kind	0	0	0	0
	Not paid	0.01	0.03	-0.01	0.04
	Does not work	0.39	0.12	0.26	0.00
Log of years of cohabiting		8.13	8	0.12	0.01
Age in years		30.19	35.4	-5.21	0
Household head		0.010	0.03	-0.03	0
Has health insurance		0.04	0.11	-0.08	0

Has savings		0.4	0.7	-0.3	0
Wealth status	Quintile 1 (poorest)	0.21	0.13	0.07	0
	Quintile 2	0.2	0.21	-0.01	0.55
	Quintile 3	0.19	0.21	-0.02	0.37
	Quintile 4	0.18	0.21	-0.04	0.05
	Quintile 5 (wealthiest)	0.23	0.23	-0.01	0.8
Ethnicity	Hausa	0.84	0.02	0.82	0
	Igbo	0.01	0.19	-0.18	0
	Yoruba	0	0.63	-0.63	0
	Others	0.04	0.17	-0.13	0
Contraceptive knowledge~~Sexual autonomy and contraceptive knowledge~~	~~Perceived lack of sexual autonomy~~	6.61~~0.42~~	9.79~~0.26~~	-3.18~~0.16~~	0~~0~~
Observations	~~Sexual autonomy~~	788~~1.01~~	865~~2.15~~	~~-1.14~~	~~0~~
	~~Contraceptive knowledge~~	~~6.61~~	~~9.79~~	~~-3.18~~	~~0~~
~~Observations~~	-	~~788~~	~~865~~	-	-

Table 2: Logit regressions: ~~Level~~ Lack of contraceptive autonomy on autonomy in household decision-making power

		(1)	(2)	(3)
State	Kano	(Reference)	(Reference)	(Reference)
	Lagos		-1.33***<br>(0.41)	-0.60<br>(0.51)
Decision-making power index		-0.10**<br>(0.05)	-0.07<br>(0.05)	-0.02<br>(0.05)
Lagos # Decision-making power index				-0.22**<br>(0.10)
Religion	Christian	(Reference)	(Reference)	(Reference)
	Islam	0.57**	0.08	0.03

		(0.25)	(0.29)	(0.30)
	Other	3.38***	3.36***	3.40***
		(0.67)	(0.66)	(0.67)
Marital Status	Currently married	(Reference)	(Reference)	(Reference)
	Currently living with partner	-0.12	-0.05	-0.07
		(0.44)	(0.44)	(0.44)
	Landowner	0.15	0.16	0.13
		(0.19)	(0.20)	(0.20)
Employment	Cash	(Reference)	(Reference)	(Reference)
	Cash and in-kind	0.41	0.32	0.24
		(0.31)	(0.32)	(0.32)
	In-kind	0.77	0.66	0.59
		(0.90)	(0.90)	(0.89)
	Not paid	0.05	0.11	-0.04
		(0.52)	(0.52)	(0.52)
	Does not work	-0.26	-0.27	-0.26
		(0.18)	(0.18)	(0.18)
Polygamous relationship		0.39**	0.31*	0.33**
		(0.16)	(0.16)	(0.16)
Log of years of cohabiting		-0.05	-0.08	-0.08
		(0.10)	(0.10)	(0.10)
Age in years		-0.00	0.00	0.00
		(0.01)	(0.01)	(0.01)
Has health insurance		0.61**	0.69**	0.74***
		(0.28)	(0.28)	(0.28)
Has savings		-0.27*	-0.23	-0.22
		(0.16)	(0.16)	(0.16)
Wealth status	Quintile 1 (poorest)	(Reference)	(Reference)	(Reference)
	Quintile 2	0.28	0.30	0.29
		(0.24)	(0.24)	(0.24)
	Quintile 3	0.61**	0.62**	0.62**
		(0.24)	(0.24)	(0.24)

	Quintile 4	0.29	0.28	0.27
		(0.26)	(0.26)	(0.26)
	Quintile 5 (wealthiest)	0.48*	0.35	0.29
		(0.26)	(0.27)	(0.27)
Ethnicity	Hausa	(Reference)	(Reference)	(Reference)
	Igbo	-0.21	0.37	0.40
		(0.37)	(0.42)	(0.42)
	Yoruba	-0.65***	0.29	0.33
		(0.24)	(0.38)	(0.37)
	Others	0.16	0.43*	0.46**
		(0.21)	(0.22)	(0.22)
Household head		0.60	0.67	0.65
		(0.48)	(0.49)	(0.50)
Contraceptive knowledge		-0.01	-0.00	-0.01
		(0.02)	(0.02)	(0.02)
Constant		-1.54**	-0.97	-1.01
		(0.72)	(0.74)	(0.75)
Observations		1,652	1,652	1,652

***p<0.01 **p<0.05 ***p<0.1

In Table 2, we report the results from the logistics regression of our contraceptive autonomy variables onto our set of covariates. In column (1), we regress lack of contraceptive autonomy ~~(LAC_1)~~ onto household decision-making power and other covariates, excluding our cultural context and institutional variables (~~religion and state~~state). ~~These factors are~~State is included in column (2). In column (3), the ~~-~~interaction between the decision-making power index and state is included to see how cultural context influences the relationship.

~~and (4), our model is the same as in column (2), except we change our dependent variables to lack of autonomy in the choice to use contraception LAC_2 (column (3)) and lack of autonomy in choice to *not* use contraception LAC_3 (column (4)).~~

The main results show that those women who ~~are joint or sole decision makers in the household~~score higher on the decision-making power index~~who are joint or sole decision makers in the household~~ are less likely to report lacking autonomy when it comes to contraceptive use (Column (1)) a relationship that is significant at the 5% level. Once the s~~S~~tate is introduced (Column (2)), the estimate of the decision-making power index is reduced and its relationship with contraceptive autonomy became statistically insignificant. However, s~~S~~tate shows a statistically significant relationship with contraceptive autonomy. Specifically, those who live in Lagos are significantly less likely to report lack of contraceptive autonomy (the relationship is significant at 1%). This suggests that institutional environment is important in the relationship between women empowerment and contraceptive autonomy. Lastly, Column (3) includes the interaction between decision-making index and state, the interaction term is statistically significant (at the 5% level), suggesting that those with higher level of decision-making power living in Lagos are less likely to report lacking contraceptive autonomy compared to women living in Kano.

When we observe the relationship between religion and contraceptive autonomy, we initially observe that being Islamic is positively and statistically significantly related to a lack of contraceptive autonomy relative to being Christian. However, the effect disappears when we control for state (columns (2) and (3)).

Figure 1: The marginal effect of the relationship between decision-making power and lack of contraceptive autonomy

Formatted: Font: Italic

Figure 1 highlight our main result by showing the marginal effect of the relationship between decision-making and lack of contraceptive autonomy. First, lack of contraceptive autonomy is higher in Kano irrespective of the level of decision-making power. However, at lower levels of the decision-making power index (0 or 1 decision out of 6), there is no significant difference between the partial effect of decision-making on contraceptive autonomy (as indicated by the overlapping confidence intervals). For women whose decision-making index ranges from 2 to 6, higher value of the decision making reduces the probability of reporting lack of contraceptive autonomy. Lastly, while the negative relationship between decision making and lack of contraceptive autonomy is apparent for women in Lagos, the relationship is flat in Kano. This suggests that for women in Kano, autonomy in other spheres of life do not necessarily translate into contraceptive autonomy.

~~. State and religion are also statistically significant predictors of contraceptive autonomy. Households from other religions are statistically significantly more likely to lack contraceptive autonomy (LAC_4) relative to households with Christian household heads (Column (2)). Living in Lagos as opposed to Kano is negatively associated with LAC_4, i.e., women living in Lagos have more contraceptive autonomy than in Kano. However, Column (2) reveals the importance of institutions and context as a potential ameliorating factor in the relationship between contraceptive autonomy and decision-making power. After controlling for state and religion, the result of being a sole or joint decision maker becomes smaller and statistically insignificant. This suggests that institutional environment is important in the relationship between women empowerment and contraceptive autonomy. The last two columns show that the negative relationship in column 1 is driven by those whose lack of autonomy results in their non-use of contraception.~~

Table 3 shows the result for those who are less likely to be involved in household decision-making. Unlike the previous result, those who less likely to be involved in household decision-making are also more likely to report lacking autonomy in contraceptive use. Consistent with the result in Table 2, column 2 show that once state is controlled for the relationship between general autonomy and contraceptive use become statistically insignificant. Lastly columns 3 and 4 show that the positive result between lack of autonomy in general and lack of autonomy is contraceptive use is driven by those whose lack of autonomy results in their non-use of contraception.

Table 3: Logit regressions: Level of contraceptive autonomy on lack of autonomy in household decision-making power

-	-	Lack of contraceptive autonomy	Lack of contraceptive autonomy	Lack of autonomy in choice to use contraception	Lack of autonomy in choice to not use contraception
Non-decision-maker		0.34**	0.22	-0.6	0.37**
		(0.16)	(0.17)	(0.38)	(0.18)
Lagos			-1.25***	0.38	-1.73***
			(0.41)	(0.71)	(0.51)
Religion of household head	Christian		(reference)	(reference)	(reference)
	Islamic		0.09	0.14	0.06
			(0.29)	(0.43)	(0.39)
	Other		3.39***	3.95***	1.86**
			(0.67)	(0.76)	(0.83)
Marital status	Currently married	(reference)	(reference)	(reference)	(reference)
	Currently living with partner	-0.14	-0.07	0.73	-0.43
		(0.43)	(0.44)	(0.6)	(0.63)
Polygamous relationship		0.39**	0.31*	0.41	0.23
		(0.16)	(0.16)	(0.32)	(0.18)

Landowner		0.11	0.17	-0.35	0.34
		(0.19)	(0.2)	(0.35)	(0.23)
Earning status	Cash	(reference)	(reference)	(reference)	(reference)
	Cash and kind	0.41	0.29	0.66	0.07
		(0.31)	(0.32)	(0.53)	(0.37)
	In-kind	0.88	0.76	2.05*	0.03
		(0.91)	(0.91)	(1.16)	(1.17)
	Not paid	0.21	0.23	0.51	0.01
		(0.52)	(0.52)	(0.78)	(0.66)
	Does not work	-0.12	-0.2	-0.4	-0.18
		(0.17)	(0.18)	(0.4)	(0.2)
Log of years of cohabiting		-0.02	-0.08	0.59**	-0.21**
		(0.09)	(0.1)	(0.28)	(0.1)
Age in years		<0.001	<0.001	-0.05	0.01
		(0.01)	(0.01)	(0.03)	(0.01)
Has health insurance		0.54**	0.69**	0.22	0.87**
		(0.27)	(0.28)	(0.42)	(0.35)
Has savings		-0.23	-0.22	0.12	-0.32*
		(0.15)	(0.16)	(0.31)	(0.18)
Wealth Quintile	Quintile 1 (poorest)	(reference)	(reference)	(reference)	(reference)
	Quintile 2	0.23	0.32	0.78	0.26
		(0.24)	(0.24)	(0.67)	(0.26)
	Quintile 3	0.58**	0.66***	1.41**	0.50*
		(0.24)	(0.24)	(0.64)	(0.26)

	Quintile 4	0.23	0.31	1.14*	0.17
		(0.25)	(0.26)	(0.67)	(0.28)
	Quintile 5 (wealthiest)	0.47*	0.41	2.07***	-0.23
		(0.26)	(0.27)	(0.64)	(0.32)
Ethnicity	Hausa	(reference)	(reference)	(reference)	(reference)
	Igbo	-0.37	0.4	-0.5	0.62
		(0.3)	(0.42)	(0.77)	(0.51)
	Yoruba	-0.69***	0.3	-0.46	0.39
		(0.23)	(0.38)	(0.7)	(0.47)
	Others	0.01	0.43*	-0.29	0.51**
		(0.2)	(0.22)	(0.61)	(0.23)
Household head		0.41	0.58	-0.3	0.91*
		(0.48)	(0.5)	(1.06)	(0.54)
Perceived lack of sexual autonomy		0.11	0.12	0.21	0.08
		(0.09)	(0.09)	(0.18)	(0.1)
Sexual autonomy		-0.14***	-0.12**	-0.01	-0.14**
		(0.05)	(0.05)	(0.09)	(0.06)
Contraceptive knowledge		-0.01	0	0.10**	-0.03
		(0.02)	(0.02)	(0.04)	(0.03)
Observations		1,652	1,652	1,652	1,652

***p<0.01 **p<0.05 ***p<0.1

Before presenting the result of the GBM model, Figs 1 and 2 show the balance statistics for the main effects presented in Table 4 (first row of Table 4). Specifically, the GBM model minimized the mean of the standardized difference in means across covariates (the Figures also shows the result when the Kolmogorov-Smirnov statistic is minimized). It is clear that the GBM weights reduce imbalance across the covariates, with the weighted standardized differences in means of all covariates falling below the 0.1 or 0.2 threshold recommended in the literature (Austin, 2009; Stuart et al., 2013). This makes the first part of the sequential ignorability assumption more plausible. Figs S1 and S2 in the supplementary material shows the balance statistics for the total effects (similar to the ones shown here) and the elements of the mediation effects (i.e., $NIE_1, NIE_0, NDE_1 and\ NDE_0$). Similar statistics for the disaggregated analysis (LAC_2 and LAC_3) are shown in the supplementary material (Figs S3-S6).

Fig 1: Balance statistics for sole and joint decision-makers (main effect)

Fig 2: Balance statistics for non-decision makers (main effect)

The results from the mediation analysis for LAC1 (contraceptive use and non-use) are reported in Table 4. For the GBM we use the following parameters, the maximum number of GBM iterations is 10, 000, the shrinkage parameter is set at 0.01 and the level of interactions allowed is 3. The substantive result is consistent with the result from the logit model, although the estimates are smaller in magnitude (suggesting the selection on observables matter). Further, the additional mediation analysis show that while institutional variables mediate the causal relationship for those who have sole or joint household decision-making power, this is not the case for those who lack household decision-making power. Specifically, NDE_1 shows that about 28 percent (-0.02/-0.07) of the total effect of decision-making power on contraceptive autonomy is via the institutional variables. While for those who lack general household autonomy the effect is direct and the effect through the mediators is not statistically

significant. This suggests that the mediators only come into play (to reinforce the direct effect) when women have decision-making power in the household.

Table 4: Household autonomy and autonomy in contraceptive use and non-use (LAC$_1$)

-	Sole or joint decision-maker				Non-decision-maker			
-	Effect	Standard Error	Confidence Interval Minimum	Confidence Interval Maximum	Effect	Standard Error	Confidence Interval Minimum	Confidence Interval Maximum
Treatment effect	**-0.07**	0.02	-0.11	-0.03	**0.05**	0.03	<0.01	0.1
Natural Direct effect - Control group NDE_0	**-0.05**	0.02	-0.1	-0.01	0.04	0.02	-0.01	0.08
Natural Indirect effect - Treatment group NIE_1	**-0.02**	0.01	-0.02	-0.01	0.01	0.01	-0.01	0.03
Natural Direct effect - Treatment group NDE_1	**-0.06**	0.02	-0.1	-0.01	**0.05**	0.03	<0.01	0.1
Natural Indirect effect - Control group NIE_0	**-0.01**	0.005	-0.02	<-0.01	0.00	0.01	-0.01	0.01

*Note: Emboldened text is statistically significant at a 5% level.

In the supplementary material (Table S1 and S2), we present the results for non-autonomous contraceptive use (LAC$_2$) and non-use (LAC$_3$). The result show similar pattern to the logit model, with non-autonomous non-use (LAC$_3$) being significant and non-autonomous use (LAC$_2$) being statistically insignificant (after controlling for selection on observables). Furthermore, for the mediation analysis, the mediation quantity of interest NIE_1 is only significant for sole/joint decision makers in the case of non-autonomous non-use.

Discussion

This paper finds evidence of a relationship between state as an institution, household decision-making power and contraceptive autonomy, controlling for selection on observables. Household decision-making power may influence contraceptive autonomy through a myriad of avenues. Firstly, decision-making power may reflect financial independence and empowerment, which has been associated with the acceptability of discussing fertility choices and family planning with a partner (Hogan, Berhanu, &

Hailemariam, 1999). Economic decision-making power and education have also been associated with lower levels of unmet contraception needs, likely through better access to health facilities (Ali & Okud, 2013). Decision-making power improves knowledge of contraception (Hogan et al., 1999), which in turn is related to the decision to use contraception (Hailemariam & Haddis, 2011; Wulifan, Brenner, Jahn, & De Allegri, 2015).

~~However, ~~T~~t~~his relationship between household decision-making power and contraceptive autonomy has to some extent been established in the literature. The unique contribution of this paper is the finding that show the overpowering role of institutions in mediating the potential impact that decision-making power can play in improving women's contraceptive autonomy. Although we find a relationship between decision-making power and contraceptive autonomy, this relationship is complicated when we control for state – a proxy for institutions. Firstly, we see that at low levels of decision-making power, there is a lack of contraceptive autonomy for women in both Lagos and Kano. However, at higher levels of decision-making power in Lagos – a state characterised by relatively higher female empowerment - women are less likely to report a lack of contraceptive autonomy. The same relationship does not hold in Kano. This suggests that for women in Kano, autonomy in other spheres of life do not necessarily translate into contraceptive autonomy.~~We firstly explore the relationship using logit regressions, and find that although there is a significant relationship between decision-making power and a lack of overall contraceptive autonomy (LAC_4), this relationship disappears when we control for state and religion. This result is found when we focus on sole or joint decision makers where there is a negative relationship with LAC_4, and non-decision-makers, where there is a positive relationship with LAC_4.~~

Secondly, novel mediation analysis is used to test whether the role of institutional variables as an ameliorating factor holds under a more rigorous empirical application. We found that institutions matter only for women who have sole or joint decision-making power. In fact, 28 percent of the total effect of sole or joint decision-making power on contraceptive autonomy is via the role of the women's state and religion. The same is not true for non-decision makers. Being a non-decision-maker directly affects women's contraceptive autonomy, regardless of their state or religion.

Our finding supports the theory that institutions play an important role in establishing the boundaries of women's autonomy, and therefore, their contraceptive autonomy. Although not oft studied, similar findings on the role of institutions have been found in other countries. In India, for instance, one study found that while her education has a positive influence on contraceptive use, the educational level and cultural norms of a woman's community plays a bigger contributing role to her contraception choice (Moursund & Kravdal, 2003). In neighbouring Ghana, Crissman *et al.* (2012) find something dissimilar. The authors find that although individually measured sexual empowerment is a positive predictor of contraceptive use, a regional average of sexual empowerment – a proxy of regional level gender norms around women's sexual empowerment– does not significantly contribute to this relationship (Crissman, Adanu, & Harlow, 2012).

The results also indicate the potential severity of household-–level disempowerment of women. Regardless of their community's adoption of specific norms and attitudes towards contraception, women who lack decision-making power in the household lack choice in various aspects of their daily existence. Autonomy in one area of life often manifests in other areas of life. As pointed out by Crissman *et al.* (2012), the multidimensional nature of empowerment – referring to empowerment across economic, political, sexual, interpersonal and socio-cultural dimensions – mean that

Formatted: Left, Line spacing: single, Don't adjust space between Latin and Asian text, Don't adjust space between Asian text and numbers

empowerment in one sphere does not always equate to empowerment in another sphere (Crissman et al., 2012).

There have been many individual level interventions to improve access to family planning services to reach women alone (F. OlaOlorun, 2010). For instance, one study in South Africa tested the feasibility of providing women with contraception and counselling at hairdressers, as it is considered a safe, community-based space which is predominantly frequented by women. Offering contraception in this space would allow women to make decisions regarding their sexual- and reproductive health independently from their partners (Bassett et al., 2018). Evidence in Zambia points to a similar strategy in improving contraceptive access. Ashraf *et al.* in a Randomised Controlled Trial tested the difference in contraceptive access when women were offered a voucher to access contraception by themselves, or when access required their partners approval. The authors found that involving spouses in family planning visits resulted in a significant drop in contraceptive access. However, the authors do note that two years after the experiment, there is a significant emotional cost to women who had made their contraception choices in isolation (Ashraf, Field, & Lee, 2014).

~~While we are unable to assess the healthcare systems and access to concealable contraception among our sample of women, we do find that there is a portion of women who are choice constrained (both in their household decision-making and in their choice not to use of contraception), and this is independent of their state and belief. Improving physical access to concealable contraception received in privacy may not be a strong enough stand-alone policy to address this level of disempowerment.~~

Limitations

Our analysis is not without limitations, importantly we note that the relationships identified are in terms of correlation between the variables of interest and may not have causal interpretation since we

are unable to control for unobserved factors. It is also important to note that all concepts are captured using self-reported measures. It is of course possible that institutions such as gender norms or sexual empowerment may affect the accuracy of reporting.

Formatted: Font: Italic

Conclusion

Institutions, culture, and context can play a key role in empowering women, emboldening their decision-making power, and enhancing their contraceptive autonomy. The paper quantifies the importance of engraining gender equality into discussions around sexual and reproductive health, even more so in contexts where specific values suppress contraceptive and reproductive autonomy. ~~Our analysis is not without limitations, importantly we note that the relationships identified are in terms of correlation between the variables of interest and may not have causal interpretation since we are unable to control for unobserved factors.~~

References

Adebowale, A. S. (2019). Ethnic disparities in fertility and its determinants in Nigeria. *Fertility research and practice, 5*(1), 1-16.

Ali, A. A. A., & Okud, A. (2013). Factors affecting unmet need for family planning in Eastern Sudan. *BMC public health, 13*(1), 1-5.

Ashraf, N., Field, E., & Lee, J. (2014). Household bargaining and excess fertility: an experimental study in Zambia. *American Economic Review, 104*(7), 2210-2237.

Bassett, I. V., Xu, A., Govere, S., Thulare, H., Frank, S. C., Psaros, C., & Parker, R. A. (2018). Contraception and PrEP in South African hair salons: Owner, stylist, and client views. *Journal of Acquired Immune Deficiency Syndromes (1999), 79*(2), e78.

Bongaarts, J. (2017). The effect of contraception on fertility: Is sub-Saharan Africa different? *Demographic Research, 37*.

Brown, S. S., & Eisenberg, L. (1995). Unintended pregnancy and the well-being of children and families. *JAMA, 274*(17), 1332-1332.

Centre for Research, E. R. a. D. C., Bayero University Kano (BUK), & The Bill & Melinda Gates Institute for Population and Reproductive Health at The Johns Hopkins Bloomberg School of Public Health.

(2020). *Performance Monitoring for Action (PMA) Household and Female Survey Phase 1, PMA/Nigeria-HQFQ-P1 (Kano & Lagos).*

Chirkov, V. (2017). Culture and autonomy. In A. T. Church (Ed.), *The Praeger Handbook of Personality Across Cultures [3 volumes]*: ABC-CLIO.

Crissman, H. P., Adanu, R. M., & Harlow, S. D. (2012). Women's sexual empowerment and contraceptive use in Ghana. *Studies in family planning, 43*(3), 201-212.

Do, M., & Kurimoto, N. (2012). Women's empowerment and choice of contraceptive methods in selected African countries. *International perspectives on sexual and reproductive health*, 23-33.

Elaigwu, J. I., & Galadima, H. (2003). The shadow of Sharia over Nigerian federalism. *Publius: The Journal of Federalism, 33*(3), 123-144.

Fadiran, D. O., Sarr, M., & Fedderke, J. W. (2017). Measurement and Analysis of the Evolution of Institutions in Nigeria. *Lagos, Nigeria: Islamic Publications Bureau.*

Hailemariam, A., & Haddis, F. (2011). Factors affecting unmet need for family planning in southern nations, nationalities and peoples region, Ethiopia. *Ethiopian journal of health sciences, 21*(2), 77-90.

Hill, N. J., Siwatu, M., & Robinson, A. K. (2014). "My religion picked my birth control": The influence of religion on contraceptive use. *Journal of religion and health, 53*, 825-833.

Hogan, D. P., Berhanu, B., & Hailemariam, A. (1999). Household organization, women's autonomy, and contraceptive behavior in southern Ethiopia. *Studies in family planning, 30*(4), 302-314.

Kandpal, E., & Baylis, K. (2019). The social lives of married women: Peer effects in female autonomy and investments in children. *Journal of development economics, 140*, 26-43.

Kendhammer, B. (2013). The Sharia controversy in Northern Nigeria and the politics of Islamic law in new and uncertain democracies. *Comparative Politics, 45*(3), 291-311.

Moursund, A., & Kravdal, Ø. (2003). Individual and community effects of women's education and autonomy on contraceptive use in India. *Population studies, 57*(3), 285-301.

Mutumba, M., Wekesa, E., & Stephenson, R. (2018). Community influences on modern contraceptive use among young women in low and middle-income countries: a cross-sectional multi-country analysis. *BMC public health, 18*(1), 1-9.

National Population Commission. (2013). *Nigeria demographic and health survey 2013*. Retrieved from

National Population Commission. (2015). *Nigerian Population Census.*

Nkoka, O., Lee, D., Chuang, K.-Y., & Chuang, Y.-C. (2021). Multilevel analysis of the role of women's empowerment on use of contraceptive methods among married Cambodian women: evidence from demographic health surveys between 2005 and 2014. *BMC Women's Health, 21*(1), 1-13.

North, D. C. (1991). Institutions. *Journal of economic perspectives, 5*(1), 97-112.

Nwadialor, K. L. (2013). Christian Missionaries and Civilization in Southern Nigeria, 1841-1960: Implications for Contemporary Christians. *UJAH: Unizik Journal of Arts and Humanities, 14*(2), 173-193.

Ojo, O. (2008). The Organization of the Atlantic Slave Trade in Yorubaland, ca. 1777 to ca. 1856. *The International journal of African historical studies, 41*(1), 77-100.

OlaOlorun, F. (2010). Advancing family planning research in Africa. *African journal of reproductive health, 14*(4).

OlaOlorun, F. M., & Hindin, M. J. (2014). Having a say matters: influence of decision-making power on contraceptive use among Nigerian women ages 35–49 years. *PloS one, 9*(6), e98702.

Ostien, P., & Dekker, A. (2010). Sharia and national law in Nigeria. *Sharia incorporated: A comparative overview of the legal systems of twelve Muslim countries in past and present*, 553-612.

Park, M. S.-A., & Chirkov, V. (2020). Culture, Self, and Autonomy. *Frontiers in Psychology, 11*, 736.

Patrikar, S., Basannar, D., & Sharma, M. S. (2014). Women empowerment and use of contraception. *Medical journal armed forces India, 70*(3), 253-256.

Pinter, B., Hakim, M., Seidman, D. S., Kubba, A., Kishen, M., & Di Carlo, C. (2016). Religion and family planning. *The European Journal of Contraception & Reproductive Health Care, 21*(6), 486-495.

Rettig, E. M., Fick, S. E., & Hijmans, R. J. (2020). The Female Empowerment Index (FEMI): spatial and temporal variation in women's empowerment in Nigeria. *Heliyon, 6*(5), e03829.

Shariff, A. F. (2015). Does religion increase moral behavior? *Current Opinion in Psychology, 6*, 108-113.

Sundararajan, R., Yoder, L. M., Kihunrwa, A., Aristide, C., Kalluvya, S. E., Downs, D. J., . . . Downs, J. A. (2019). How gender and religion impact uptake of family planning: results from a qualitative study in Northwestern Tanzania. *BMC Women's Health, 19*, 1-10.

The World Bank. (2022a). Fertility rates, total (births) per woman. Retrieved from https://data.worldbank.org/indicator/SP.DYN.TFRT.IN?locations=NG

The World Bank. (2022b). Population, total - Nigeria. Retrieved from https://data.worldbank.org/indicator/SP.POP.TOTL?locations=NG

Woldemicael, G. (2009). Women's autonomy and reproductive preferences in Eritrea. *Journal of biosocial science, 41*(2), 161-181.

Wulifan, J. K., Brenner, S., Jahn, A., & De Allegri, M. (2015). A scoping review on determinants of unmet need for family planning among women of reproductive age in low and middle income countries. *BMC Women's Health, 16*(1), 1-15.

Yaya, S., Uthman, O. A., Ekholuenetale, M., & Bishwajit, G. (2018). Women empowerment as an enabling factor of contraceptive use in sub-Saharan Africa: a multilevel analysis of cross-sectional surveys of 32 countries. *Reproductive health, 15*(1), 1-12.

Article: Institutions matter: The role of institutions in the relationship between decision-making power and contraceptive autonomy
Manuscript Number: PGPH-D-23-02195

We thank the reviewer for the helpful comments. After reflecting on the comment about our methodology, specifically the use of mediation analysis, we agree with the reviewer that this is not the appropriate method. This is mainly because the temporal ordering assumption (i.e. autonomy influencing location) is problematic. Since our main aim is to investigate how institutions influence the relationship between autonomy and contraceptive use, we think of location as a confounder as suggested in the review comment. Specifically, we argue for the role of institution in the relationship between general autonomy and autonomy in contraceptive use based on institutional theory that argues that institutions shape individuals' behaviours and interactions within a society. Our main hypothesis in the revised draft is that institution is a confounder in the sense that the relationship between general autonomy and contraceptive autonomy depends on institutional context. We operationalize this by interacting general autonomy with our institution proxy (state) in our model to show that relationship between general autonomy and contraceptive autonomy depends on institutional environment.

We also consider multilevel modelling as suggested, however because we have only 2 clusters, we did not think this is viable. Therefore, we have presented result based on the interaction model in the revised draft.

Review Comments to the Authors:
This study applies a novel method to explore the role of institutions in the relationship between women's decision-making power and contraceptive autonomy. While there are numerous strengths to this endeavor, I advise authors to include justification that the assumptions for causal mediation analysis are met. Alternatively, authors may want to consider other methods, such as structural equation modeling or multilevel models.

Study Clarifications

1. It would be helpful to ground research and methods with a theoretical or conceptual framework. As none is specifically mentioned, it is more difficult to follow along the application of these methods and selected covariates in addressing research objectives.

Response: Thank you to the reviewer for the comment. We have made it clear that our analysis is based on institutional theory, and that this forms the basis of our conceptual framework. Please see the introduction of the paper. We have rewritten and shifted the entire introduction using track changes to highlight this conceptual framework.

2. The causal inference framework in mediation research are most frequently applied to answering questions such as, "If we could fix the mediator at a specific value, how would that change outcomes and what would be the effect of exposure on outcome?" (direct effects) or "What would be the effect of exposure on outcome if we could block the direct path from exposure to mediator in some manner?" (indirect effects). This raises the question on the selection of location of residence (Kano and Lagos) as a Comment 1 mediator (vs confounder); and Comment 2 that causal mediation analysis is appropriate.

a. Comment 1: For instance, where one lives is not random and is subject to compositional or social selection, and as mentioned by the authors, the two cities differ by deep-rooted historical, cultural, and kinship factors. This mechanism sorts people into where they live as well as influences the opportunities and appropriate behaviors (such as contraceptive autonomy). Authors should add justification for decisions in treating residence a mediator rather than a confounder given that residence is associated norms and behaviors around decision making in the household and contraceptive autonomy. Referencing a theory/framework regarding contraceptive autonomy/women's autonomy/empowerment etc. would be a great start.

b. Comment 2: For instance, authors may need to consider and discuss the plausibility that the foundational assumptions to mediation analyses are met to assess identifiability:

   i. Temporal ordering

   ii. Confounder adjustment (selected covariates are appropriate, however analyses should explain or provide evidence for which relationships were deemed confounded to justify assumptions of

      1. No unmeasured exposure–outcome;
      2. No mediator–outcome confounding;
      3. No unmeasured exposure–mediator confounding;
      4. No post-treatment confounders

   iii. Consistency

   iv. Positivity

   v. If authors are concerned that an identification assumption may not hold, then authors should include sensitivity analyses to demonstrate results hold sure with added assumption uncertainty.

Response: As indicated above we have removed the previous model that presented mediation analysis since this is not consistent with the assumption that validates the model.

Comments

3. **Methods:** Please make it clear how mediation effects were estimated using GBM results (i.e. weighted regression)

Response: Please see our response on methodology.

4. **Method**: Please re-mention or clarify the covariates included in the matching process (making sure to not match on covariates that are outcomes of the exposure)

Response: Thank you for this comment, we have re-examined the covariates and removed two of the covariates that relate to sexual autonomy as this can be argued to be outcomes rather than covariates.

5. **Methods:** Please provide the statistical software and any packages used for these analyses.

Response: Our initial analysis is performed using the "twangMediation" package in R but the revised analysis is based on Stata. This has been noted in the manuscript.

6. **Methods:** Description of sensitivity analysis done to assess sensitivity to unobserved confounding in propensity score matching.

Response: This is not necessary given our change in methodology.

7. **Results:** Propensity score covariate balance: Figures 1 and 2 show the balance statistics for covariates after GBM, authors should include labels for covariates in the figure or consider providing pre- post- standardized differences in table. Please make sure that figure labels are informative and any abbreviations are written out in table footnotes.

Response: Please see out response on methodology

8. **Discussion**: Please elaborate on study limitations, such as in a separate paragraph.

Response: Thank you for the suggestion. We have created a study limitations section as the last paragraph of the discussion. We point out the cross-sectional nature of our analysis, as well as the potential reporting bias due to the nature of the topic.

9. **Discussion**: Comparison of findings with the literature. Particularly to support claims in paragraph lines 390-403.

Suggested helpful references:
- Trang et al. (2022). https://doi.org/10.48550/arXiv.2011.09537
- Vanderweele, Vansteelandt & Robins. (2015). https://doi.org/10.1097%2FEDE.0000000000000034

Response: We agree with the recommendation. Our discussion has changed quite substantially due to the changing of our methodology, but we've added country and study comparisons throughout. For instance, the following was added to line 487:

"Our finding supports the theory that institutions play an important role in establishing the boundaries of women's autonomy, and therefore, their contraceptive autonomy. Although not oft studied, similar findings on the role of institutions have been found in other countries. In India, for instance, one study found that while her education has a positive influence on contraceptive use, the educational level and cultural norms of a woman's community plays a bigger contributing role to her contraception choice (Moursund & Kravdal, 2003). In neighbouring Ghana, Crissman *et al.* (2012) find something dissimilar. The authors find that although individually measured sexual empowerment is a positive predictor of contraceptive use, a regional average of sexual empowerment – a proxy of regional level gender norms around women's sexual empowerment– does not significantly contribute to this relationship (Crissman, Adanu, & Harlow, 2012)."
