# Supplementary material for: Institutions matter: The role of institutions in the relationship between decision-making power and contraceptive autonomy
Source: PLOS Glob Public Health. 2024 Nov 13;4(11):e0003913. doi: 10.1371/journal.pgph.0003913 (PMC11560016; doi:10.1371/journal.pgph.0003913)
Supplement: Attachment — Submitted filename: PGPH-D-23-02195_Review.docx [file pgph.0003913.s001.docx]

**Article**: Institutions matter: The role of institutions in the relationship between decision-making power and contraceptive autonomy

**Manuscript Number:** PGPH-D-23-02195

**Review Comments to the Authors**:

This study applies a novel method to explore the role of institutions in the relationship between women’s decision-making power and contraceptive autonomy. While there are numerous strengths to this endeavor, I advise authors to include justification that the assumptions for causal mediation analysis are met. Alternatively, authors may want to consider other methods, such as structural equation modeling or multilevel models.

**Study Clarifications**

1. It would be helpful to ground research and methods with a theoretical or conceptual framework. As none is specifically mentioned, it is more difficult to follow along the application of these methods and selected covariates in addressing research objectives.
2. The causal inference framework in mediation research are most frequently applied to answering questions such as, “If we could fix the mediator at a specific value, how would that change outcomes and what would be the effect of exposure on outcome?” (direct effects) or “What would be the effect of exposure on outcome if we could block the direct path from exposure to mediator in some manner?” (indirect effects). This raises the question on the selection of location of residence (Kano and Lagos) as a Comment 1 mediator (vs confounder); and Comment 2 that causal mediation analysis is appropriate.
   a. Comment 1: For instance, where one lives is not random and is subject to compositional or social selection, and as mentioned by the authors, the two cities differ by deep-rooted historical, cultural, and kinship factors. This mechanism sorts people into where they live as well as influences the opportunities and appropriate behaviors (such as contraceptive autonomy). Authors should add justification for decisions in treating residence a mediator rather than a confounder given that residence is associated norms and behaviors around decision making in the household and contraceptive autonomy. Referencing a theory/framework regarding contraceptive autonomy/women’s autonomy/empowerment etc. would be a great start.
   b. Comment 2: For instance, authors may need to consider and discuss the plausibility that the foundational assumptions to mediation analyses are met to assess identifiability:
      i. Temporal ordering
      ii. Confounder adjustment (selected covariates are appropriate, however analyses should explain or provide evidence for which relationships were deemed confounded to justify assumptions of
          1. No unmeasured exposure–outcome;
          2. No mediator–outcome confounding;
          3. No unmeasured exposure–mediator confounding;
          4. No post-treatment confounders
      iii. Consistency
      iv. Positivity
      v. If authors are concerned that an identification assumption may not hold, then authors should include sensitivity analyses to demonstrate results hold sure with added assumption uncertainty.

**Comments**

3. **Methods:** Please make it clear how mediation effects were estimated using GBM results (i.e. weighted regression)
4. **Method**: Please re-mention or clarify the covariates included in the matching process (making sure to not match on covariates that are outcomes of the exposure)
5. **Methods:** Please provide the statistical software and any packages used for these analyses.
6. **Methods:** Description of sensitivity analysis done to assess sensitivity to unobserved confounding in propensity score matching.
7. **Results:** Propensity score covariate balance: Figures 1 and 2 show the balance statistics for covariates after GBM, authors should include labels for covariates in the figure or consider providing pre- post- standardized differences in table. Please make sure that figure labels are informative and any abbreviations are written out in table footnotes.
8. **Discussion**: Please elaborate on study limitations, such as in a separate paragraph.
9. **Discussion**: Comparison of findings with the literature. Particularly to support claims in paragraph lines 390-403.

Suggested helpful references:

- Trang et al. (2022). <https://doi.org/10.48550/arXiv.2011.09537>
- Vanderweele, Vansteelandt & Robins. (2015). <https://doi.org/10.1097%2FEDE.0000000000000034>
