# Supplementary material for: Institutions matter: The role of institutions in the relationship between decision-making power and contraceptive autonomy
Source: PLOS Glob Public Health. 2024 Nov 13;4(11):e0003913. doi: 10.1371/journal.pgph.0003913 (PMC11560016; doi:10.1371/journal.pgph.0003913)
Supplement: Attachment — Submitted filename: PGPH-D-23-02195_Response to Reviewer April 2024.docx [file pgph.0003913.s002.docx]

**Article**: Institutions matter: The role of institutions in the relationship between decision-making power and contraceptive autonomy

**Manuscript Number:** PGPH-D-23-02195

We thank the reviewer for the helpful comments. After reflecting on the comment about our methodology, specifically the use of mediation analysis, we agree with the reviewer that this is not the appropriate method. This is mainly because the temporal ordering assumption (i.e. autonomy influencing location) is problematic. Since our main aim is to investigate how institutions influence the relationship between autonomy and contraceptive use, we think of location as a confounder as suggested in the review comment. Specifically, we argue for the role of institution in the relationship between general autonomy and autonomy in contraceptive use based on institutional theory that argues that institutions shape individuals’ behaviours and interactions within a society. Our main hypothesis in the revised draft is that institution is a confounder in the sense that the relationship between general autonomy and contraceptive autonomy depends on institutional context. We operationalize this by interacting general autonomy with our institution proxy (state) in our model to show that relationship between general autonomy and contraceptive autonomy depends on institutional environment.

We also consider multilevel modelling as suggested, however because we have only 2 clusters, we did not think this is viable. Therefore, we have presented result based on the interaction model in the revised draft.

**Review Comments to the Authors**:

This study applies a novel method to explore the role of institutions in the relationship between women’s decision-making power and contraceptive autonomy. While there are numerous strengths to this endeavor, I advise authors to include justification that the assumptions for causal mediation analysis are met. Alternatively, authors may want to consider other methods, such as structural equation modeling or multilevel models.

**Study Clarifications**

1. It would be helpful to ground research and methods with a theoretical or conceptual framework. As none is specifically mentioned, it is more difficult to follow along the application of these methods and selected covariates in addressing research objectives.

Response: Thank you to the reviewer for the comment. We have made it clear that our analysis is based on institutional theory, and that this forms the basis of our conceptual framework. Please see the introduction of the paper. We have rewritten and shifted the entire introduction using track changes to highlight this conceptual framework.

2. The causal inference framework in mediation research are most frequently applied to answering questions such as, “If we could fix the mediator at a specific value, how would that change outcomes and what would be the effect of exposure on outcome?” (direct effects) or “What would be the effect of exposure on outcome if we could block the direct path from exposure to mediator in some manner?” (indirect effects). This raises the question on the selection of location of residence (Kano and Lagos) as a Comment 1 mediator (vs confounder); and Comment 2 that causal mediation analysis is appropriate.
   a. Comment 1: For instance, where one lives is not random and is subject to compositional or social selection, and as mentioned by the authors, the two cities differ by deep-rooted historical, cultural, and kinship factors. This mechanism sorts people into where they live as well as influences the opportunities and appropriate behaviors (such as contraceptive autonomy). Authors should add justification for decisions in treating residence a mediator rather than a confounder given that residence is associated norms and behaviors around decision making in the household and contraceptive autonomy. Referencing a theory/framework regarding contraceptive autonomy/women’s autonomy/empowerment etc. would be a great start.
   b. Comment 2: For instance, authors may need to consider and discuss the plausibility that the foundational assumptions to mediation analyses are met to assess identifiability:
      i. Temporal ordering
      ii. Confounder adjustment (selected covariates are appropriate, however analyses should explain or provide evidence for which relationships were deemed confounded to justify assumptions of
          1. No unmeasured exposure–outcome;
          2. No mediator–outcome confounding;
          3. No unmeasured exposure–mediator confounding;
          4. No post-treatment confounders
      iii. Consistency
      iv. Positivity
      v. If authors are concerned that an identification assumption may not hold, then authors should include sensitivity analyses to demonstrate results hold sure with added assumption uncertainty.

Response: As indicated above we have removed the previous model that presented mediation analysis since this is not consistent with the assumption that validates the model.

**Comments**

3. **Methods:** Please make it clear how mediation effects were estimated using GBM results (i.e. weighted regression)

Response: Please see our response on methodology.

4. **Method**: Please re-mention or clarify the covariates included in the matching process (making sure to not match on covariates that are outcomes of the exposure)

Response: Thank you for this comment, we have re-examined the covariates and removed two of the covariates that relate to sexual autonomy as this can be argued to be outcomes rather than covariates.

5. **Methods:** Please provide the statistical software and any packages used for these analyses.

Response: Our initial analysis is performed using the “twangMediation” package in R but the revised analysis is based on Stata. This has been noted in the manuscript.

6. **Methods:** Description of sensitivity analysis done to assess sensitivity to unobserved confounding in propensity score matching.

Response: This is not necessary given our change in methodology.

7. **Results:** Propensity score covariate balance: Figures 1 and 2 show the balance statistics for covariates after GBM, authors should include labels for covariates in the figure or consider providing pre- post- standardized differences in table. Please make sure that figure labels are informative and any abbreviations are written out in table footnotes.

Response: Please see out response on methodology

8. **Discussion**: Please elaborate on study limitations, such as in a separate paragraph.

Response: Thank you for the suggestion. We have created a study limitations section as the last paragraph of the discussion. We point out the cross-sectional nature of our analysis, as well as the potential reporting bias due to the nature of the topic.

9. **Discussion**: Comparison of findings with the literature. Particularly to support claims in paragraph lines 390-403.

Suggested helpful references:

- Trang et al. (2022). <https://doi.org/10.48550/arXiv.2011.09537>
- Vanderweele, Vansteelandt & Robins. (2015). <https://doi.org/10.1097%2FEDE.0000000000000034>

Response: We agree with the recommendation. Our discussion has changed quite substantially due to the changing of our methodology, but we’ve added country and study comparisons throughout. For instance, the following was added to line 487:

“Our finding supports the theory that institutions play an important role in establishing the boundaries of women’s autonomy, and therefore, their contraceptive autonomy. Although not oft studied, similar findings on the role of institutions have been found in other countries. In India, for instance, one study found that while her education has a positive influence on contraceptive use, the educational level and cultural norms of a woman’s community plays a bigger contributing role to her contraception choice (Moursund & Kravdal, 2003). In neighbouring Ghana, Crissman *et al.* (2012) find something dissimilar. The authors find that although individually measured sexual empowerment is a positive predictor of contraceptive use, a regional average of sexual empowerment – a proxy of regional level gender norms around women’s sexual empowerment– does not significantly contribute to this relationship (Crissman, Adanu, & Harlow, 2012).”
