# Supplementary material for: Institutions matter: The role of institutions in the relationship between decision-making power and contraceptive autonomy
Source: PLOS Glob Public Health. 2024 Nov 13;4(11):e0003913. doi: 10.1371/journal.pgph.0003913 (PMC11560016; doi:10.1371/journal.pgph.0003913)
Supplement: Attachment — Submitted filename: Response to reviewer_October 2024.docx [file pgph.0003913.s004.docx]

**Response to reviewer: “Institutions matter: The role of institutions in the relationship between decision-making power and contraceptive autonomy”**

We would like to thank the reviewer for their comments on improving the paper. Please find responses to the reviewer comments made directly in the text.

- **Reviewer comment: Page 2**

**Author response:** Thank you to the reviewer for the comment. We have removed the sentence from the manuscript.

- **Reviewer comment: Page 3**

**Author response:** Thank you to the reviewer for this comment, and we agree that “access” is a multi-faceted concept that needs to be defined. We have made two changes to the text:

1) In the paragraph prior to this comment, we have added examples of modern contraception. The sentence now reads: “A key predictor of fertility includes access to modern contraception **(Bongaarts, 2017), where modern contraceptive methods include the pill, IUD, injectables, implants, condoms, emergency contraception and male and female sterilization.”**
2) Second, after the highlighted text, we have included the following text in bold: “Access to modern contraceptive methods is a function of several structural, cultural, and socio-economic factors, including women’s empowerment and household bargaining power (Do & Kurimoto, 2012; Nkoka, Lee, Chuang, & Chuang, 2021; F. M. OlaOlorun & Hindin, 2014; Patrikar, Basannar, & Sharma, 2014; Yaya, Uthman, Ekholuenetale, & Bishwajit, 2018). **Access to modern contraception not only encompasses the physical availability of these methods, but also their acceptability and affordability.”**

- **Reviewer comment: Page 4**

**Author response:** Thank you to the reviewer for pointing this out. We are indeed referring to use rather than access. This has been changed in the manuscript.

- **Reviewer comment: Page 7**

**Author response:** We have removed “during the nineties” as the reference to the shift after the 1994 conference should suffice as an indication of the timing of the change of definition.

- **Reviewer comment: Page 10**

**Author response:** The sentence has been removed based on the comments from another reviewer.

- **Reviewer comment: Page 28**

**Author response:** These have all been removed based on another reviewer’s comments.

- **Reviewer comment: Page 33**

**Author response:** we have changed the word to “establishing”.

- **Reviewer comment:** Thank you for addressing previous recommendations. Please further expand on the discussion and/or conclusion sections to include discussion of the generalizability of the findings and recommendations for further research. Please note, too, several confusing word choices and sentence structure choices, highlighted in the attached.

**Author response:** We have addressed the comment by adding the following to the discussion section of the paper:

“Our study uses the heterogeneity of norms and religious beliefs across Nigerian states to test the role of this variability on contraceptive autonomy, the findings of which could be relevant to similar contexts in other Sub-Saharan African countries with comparable gender norms, legal systems and religious influences. The generalizability of these findings are limited by several factors. The study focuses on an urban context and the findings might not apply to decision-making power and contraceptive autonomy in more rural contexts, where the availability of healthcare services and contraception may differ. It is also unlikely that these findings are applicable in more egalitarian or secular societies, especially when several policies have been implemented to address gender inequalities.

Future research should be aimed at establishing the robustness of this relationship. This would include applying causal estimation techniques or adopting a more longitudinal approach. A qualitative approach can also be used to get a more in-depth understanding of the interplay between decision-making power, gender norms, religious beliefs and contraceptive autonomy. In addition, by including men in the data collection, and researching their attitudes towards contraceptive use in the same context, one could gain a complementary view of the current results.”

In addition, we have made all the suggested changes to sentence structures and word choices as made by the reviewer in the text.
